# Regulation of pollen lipid body biogenesis by MAP kinases and downstream WRKY transcription factors in *Arabidopsis*

**DOI:** 10.1371/journal.pgen.1007880

**Published:** 2018-12-26

**Authors:** Yueping Zheng, Xiangxiong Deng, Aili Qu, Mengmeng Zhang, Yuan Tao, Liuyi Yang, Yidong Liu, Juan Xu, Shuqun Zhang

**Affiliations:** 1 State Key Laboratory of Plant Physiology and Biochemistry, College of Life Sciences, Zhejiang University, Hangzhou, Zhejiang, China; 2 Division of Biochemistry, Interdisciplinary Plant Group, Bond Life Sciences Center, University of Missouri, Columbia, MO, United States of America; University of Massachusetts at Amherst, UNITED STATES

## Abstract

Signaling pathways that control the activities in non-photosynthetic plastids, important sites of plant metabolism, are largely unknown. Previously, we demonstrated that WRKY2 and WRKY34 transcription factors play an essential role in pollen development downstream of mitogen-activated protein kinase 3 (MPK3) and MPK6 in *Arabidopsis*. Here, we report that *GLUCOSE-6-PHOSPHATE/PHOSPHATE TRANSLOCATOR 1* (*GPT1*) is a key target gene of WRKY2/WRKY34. GPT1 transports glucose-6-phosphate (Glc6P) into plastids for starch and/or fatty acid biosynthesis depending on the plant species. Loss of function of *WRKY2/WRKY34* results in reduced *GPT1* expression, and concomitantly, reduced accumulation of lipid bodies in mature pollen, which leads to compromised pollen viability, germination, pollen tube growth, and male transmission in *Arabidopsis*. Pollen-specific overexpression of *GPT1* rescues the pollen defects of *wrky2 wrky34* double mutant. Furthermore, gain-of-function activation of MPK3/MPK6 enhances *GPT1* expression; whereas *GPT1* expression is reduced in *mkk4 mkk5* double mutant. Together, this study revealed a cytoplasmic/nuclear signaling pathway capable of coordinating the metabolic activities in plastids. High-level expression of *GPT1* at late stages of pollen development drives Glc6P from cytosol into plastids, where Glc6P is used for fatty acid biosynthesis, an important step of lipid body biogenesis. The accumulation of lipid bodies during pollen maturation is essential to pollen fitness and successful reproduction.

## Author summary

Plastids are important sites of plant metabolism including fatty acid and starch biosynthesis. At present, how the activities in the plastids are coordinated with those in the cytoplasm and the signaling pathway(s) involved are largely unknown. Previously, we demonstrated that WRKY2 and WRKY34 transcription factors play an essential role in pollen development downstream of mitogen-activated protein kinase 3 (MPK3) and MPK6 in *Arabidopsis*. Here, we report that *GLUCOSE-6-PHOSPHATE/PHOSPHATE TRANSLOCATOR 1* (*GPT1*) is a key target gene of WRKY2/WRKY34. GPT1 is localized on the membrane of plastids and transports glucose-6-phosphate (Glc6P) into plastids for starch and/or fatty acid biosynthesis depending on the plant species. Genetic analyses demonstrated that *WRKY2/WRKY34* and their upstream MPK3/MPK6 are involved in regulating *GPT1* expression, therefore, the accumulation of lipid bodies in mature pollen, which is critical to pollen viability, pollen germination, pollen tube growth, and male transmission in *Arabidopsis*. This study revealed a cytoplasmic/nuclear signaling pathway capable of coordinating the metabolic activities in plastids. High-level expression of *GPT1* at late stages of pollen development drives Glc6P from cytosol into plastids, where Glc6P is used for fatty acid biosynthesis, an important step of lipid body biogenesis. The accumulation of lipid bodies during pollen maturation is essential to pollen fitness and successful reproduction.

## Introduction

Plastids including those that are non-photosynthetic are important sites of metabolism in plants. How the metabolic pathways in plastids and those outsides are coordinated is not well understood. Pollen, the male gametophyte, is critical to reproductive success of all flowering plants [1, 2]. Development of the heterotrophic pollen requires energy and carbon inputs throughout the whole process [3, 4]. At early stages, microspore is immersed in locular fluid containing nutrients from the sporophytic tapetal cells. Later, pollen maturation requires the accumulation of carbohydrates in the forms of starch and/or lipids [5, 6]. Nutrient filling during pollen maturation is important to successful fertilization because pollen germination and pollen tube growth (at least at the early stage) are dependent on the storage compounds for carbon/energy sources [7–9]. Furthermore, stress-induced male sterility is frequently associated with the lack of storage compounds [10, 11]. As a result, understanding the regulation of nutrient accumulation during pollen maturation is important to agriculture production.

In *Arabidopsis*, mature pollen contains mostly lipid bodies, although starch is present in the vegetative cell at the early stages of pollen development [12, 13]. Lipid body biogenesis in pollen is analogous to the formation of storage oil bodies in oil seeds [14, 15], which involves two important steps that occur in different organelles. The first step is the *de novo* biosynthesis of fatty acids in plastids, which produces acyl-CoA using carbon from source materials. The second step occurs in specialized endoplasmic reticulum (ER) where acyl-CoA is added to glycerol-3-phosphate (G3P) to form triglycerides [16–20]. In pollen, fatty acid biosynthesis in the non-photosynthetic plastids relies on the import of carbon sources carried out by a number of sugar transporters including glucose-6-phosphate/phosphate translocator (GPT) isoforms that mediate the import of glucose-6-phosphate (Glc6P) into plastids [21–26]. At present, it is unclear how these different steps are coordinated in a spatiotemporal-specific manner. It is also unclear which step is the rate-limiting step in lipid body biogenesis during pollen development.

Mitogen-activated protein kinase (MAPK, or MPK) cascades are highly conserved signaling modules in eukaryotes [27–33]. MPK3/MPK6, two MAPKs among the 20 MAPKs in *Arabidopsis*, are involved in a number of growth and developmental processes by receiving signals from different receptors/sensors [31]. The multi-functionality of MPK3/MPK6 can also be attributed to the spatiotemporal-specific phosphorylation of MAPK substrates. For instance, MPK3/MPK6 are able to phosphorylate multiple WRKY transcription factors. Depending on the cell/tissue-specific expression of these WRKYs, MPK3/MPK6 carry out unique functions in different cells/tissues/organs. In vegetative tissues/organs such as leaves, phosphorylation of WRKY33 by MPK3/MPK6 regulates phytoalexin biosynthesis in plant immunity [34]. In developing pollen, MPK3/MPK6 phosphorylate WRKY34, and possibly WRKY2, in a spatiotemporal-specific manner to regulate pollen development. Mutation of both *WRKY2* and *WRKY34* resulted in defective pollen development, germination, and pollen tube growth [35].

In this report, we demonstrate that the reduced viability and transmission of *wrky2 wrky34* double mutant pollen is a result of the lack of or reduced levels of lipids, the main storage compounds in Arabidopsis pollen. WRKY2 and WRKY34 regulate the temporal expression of *GPT1*, which is essential to the lipid body accumulation during pollen development. This study revealed an important cytoplasmic/nuclear signaling pathway capable of coordinating the metabolic activities in plastids and other parts of the cells. In addition, it demonstrated that Glc6P is the key source carbohydrate for lipid body biogenesis in pollen of Arabidopsis. High-level expression of *GPT1* at the late stages of pollen development drives Glc6P from cytosol into plastids, where Glc6P is used for fatty acid biosynthesis, an important step of lipid body biogenesis. The accumulation of lipid bodies during pollen maturation is essential to pollen fitness and successful reproduction.

## Results

### Double *wrky2 wrky34* mutant pollen has reduced storage lipid bodies, similar to *gpt1* mutant pollen

**Fig 1:**
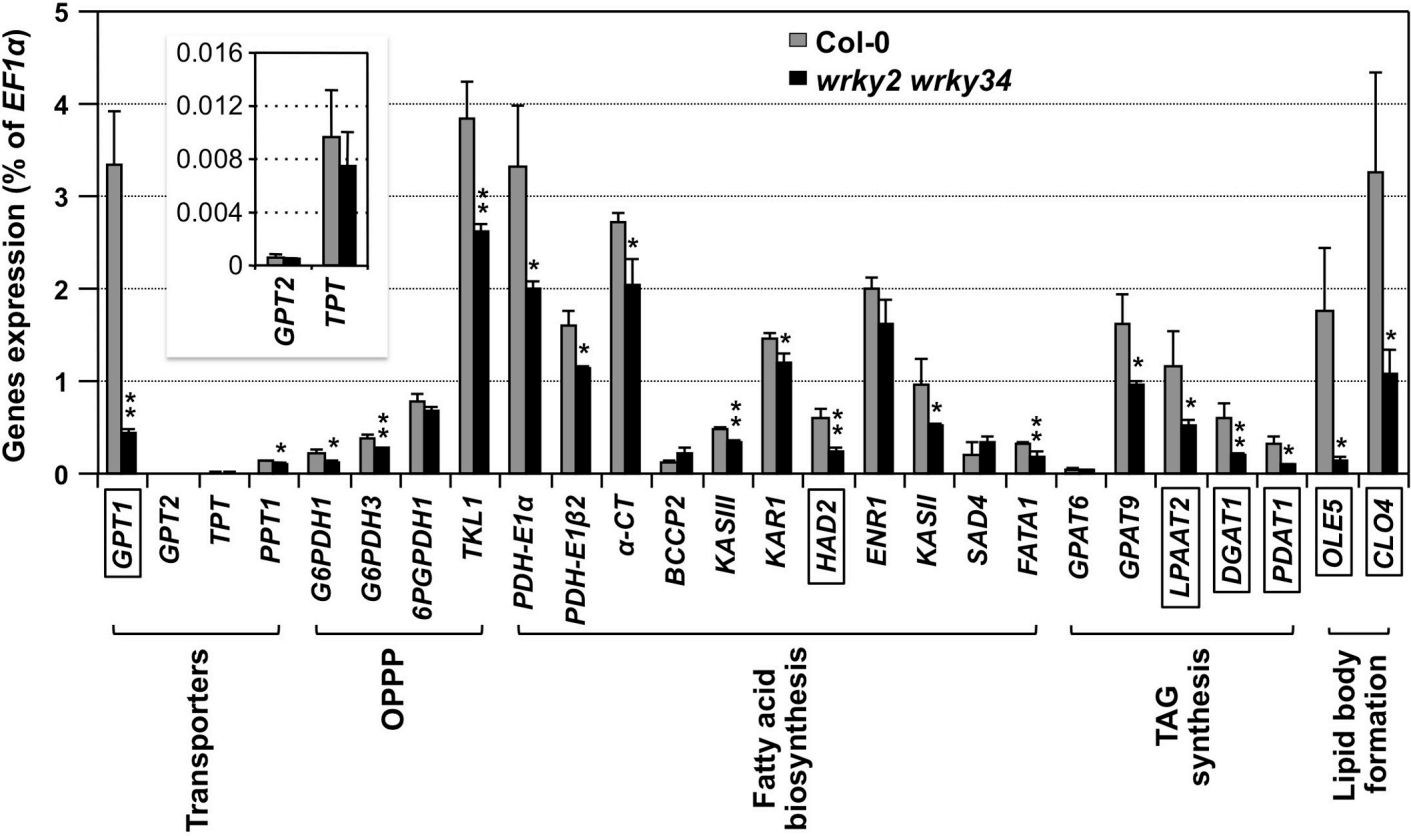
Expression of *GPT1* is compromised in *wrky2 wrky34* double mutant. Total RNAs were extracted from BCP/TCP-stage pollen grains of wild-type (Col-0) and *wrky2 wrky34* double mutant plants. After reverse transcription, expression of genes involved in lipid body biogenesis was quantitated using real-time PCR. *EF1α* was used as an internal control. Values are means ± SD, n = 3. *GPT*, glucose-6-phosphate/phosphate translocator; *TPT*, triose phosphate/phosphate translocator; *PPT*, phosphoenolpyruvate/phosphate translocator; *G6PDH*, glucose-6-phosphate dehydrogenase; *6PGPDH*, gluconate-6-phosphate dehydrogenase; *TKL*, transketolase; *PDH-E1α*, pyruvate dehydrogenase E1α subunit; *PDH-E1β2*, pyruvate dehydrogenase E1β 2 subunit; *α-CT*, α-carboxyltransferase of heteromeric ACCase; *BCCP2*, biotin carboxyl carrier protein of heteromeric ACCase 2; *KAS III*, β-ketoacyl-ACP synthase III; *KAR*, β-ketoacyl-ACP-reductase; *HAD*, β-hydroxyacyl-ACP dehydrase; *ENR*, enoyl-ACP reductase; *KAS II*, β-ketoacyl-ACP synthase II; *SAD*, stearoyl-ACP desaturase; *FATA*, fatty acyl-ACP thioesterase; *GPAT*, glycerol-3-phosphate acyltransferase; *LPAAT*, lysophosphatidic acid acyltransferase; *DGAT*, diacylglycerol acyltransferase; *PDAT*, phospholipid:diacylglycerol acyltransferase; *OLE*, oleosin; *CLO*, caleosin. OPPP, oxidative pentose phosphate pathway. TAG, triacylglycerol. Error bars indicate SD (n = 3). *P ≤ 0.05 and **P ≤ 0.01. Genes whose expression levels reduced by more than 50% in the *wrky2 wrky34* double mutant are boxed. Inset shows the expression levels of *GPT2* and *TPT* on a different scale.

We previously reported that mutation of both *WRKY2* and *WRKY34* greatly reduces pollen viability, which is associated with decreased pollen germination, pollen tube growth, and male transmission [35]. A more careful examination of the transmission electron microscopic (TEM) images (Fig 5L and 5M in [35]) revealed a reduced number of oil bodies and more void spaces in *wrky2 wrky34* double mutant pollen (Note: Oil bodies were mislabeled as plastids in the images. Plastids are double membrane-bound organelles with simple membrane structures inside. In contrast, lipid bodies have a homogenous neutral lipid core bound by a phospholipid monolayer with oleosins.), suggesting that WRKY2/WRKY34 might be involved in regulating lipid biosynthesis during pollen maturation. As a result, we compared the expression of genes encoding important enzymes/proteins related to lipid body biogenesis in pollen grains of *wrky2 wrky34* double mutant and wild type using quantitative RT-PCR. We found that the expression of *GPT1* (encoding glucose-6-phosphate/phosphate translocator 1), *HAD2* (encoding β-hydroxyacyl-ACP dehydrase 2), *LPAAT2* (encoding lysophosphatidic acid acyltransferase 2), *DGAT1* (encoding diacylglycerol acyltransferase 1), *PDAT1* (encoding phospholipid:diacylglycerol acyltransferase 1), *OLE5* (encoding oleosin 5), and *CLO4* (encoding caleosin 4) was reduced by at least 50% in *wrky2 wrky34* mutant pollen. In contrast, the expression of other genes was less affected (Fig 1).

A literature search revealed that, similar to *wrky2 wrky34* double mutant, mutation of *GPT1* also results in pollen defects including reduced lipid bodies [36]. GPTs carry out the transportation of Glc6P into plastids where Glc6P can be used for starch biosynthesis, fatty acid biosynthesis, or NADPH generation via the oxidative pentose phosphate pathway (OPPP) [21, 24, 36, 37]. Of the two GPT isoforms in *Arabidopsis*, *GPT1* gene expression was severely reduced in the pollen of *wrky2 wrky34* double mutant plants. The expression of *GPT2* was very low in pollen, but detectable using RT-qPCR. Its expression was not altered in *wrky2 wrky34* mutant (Fig 1, inset). For these reasons, we set out to test whether *GPT1* is a target gene of WRKY2/WRKY34 transcription factors during pollen development.

**Fig 2:**
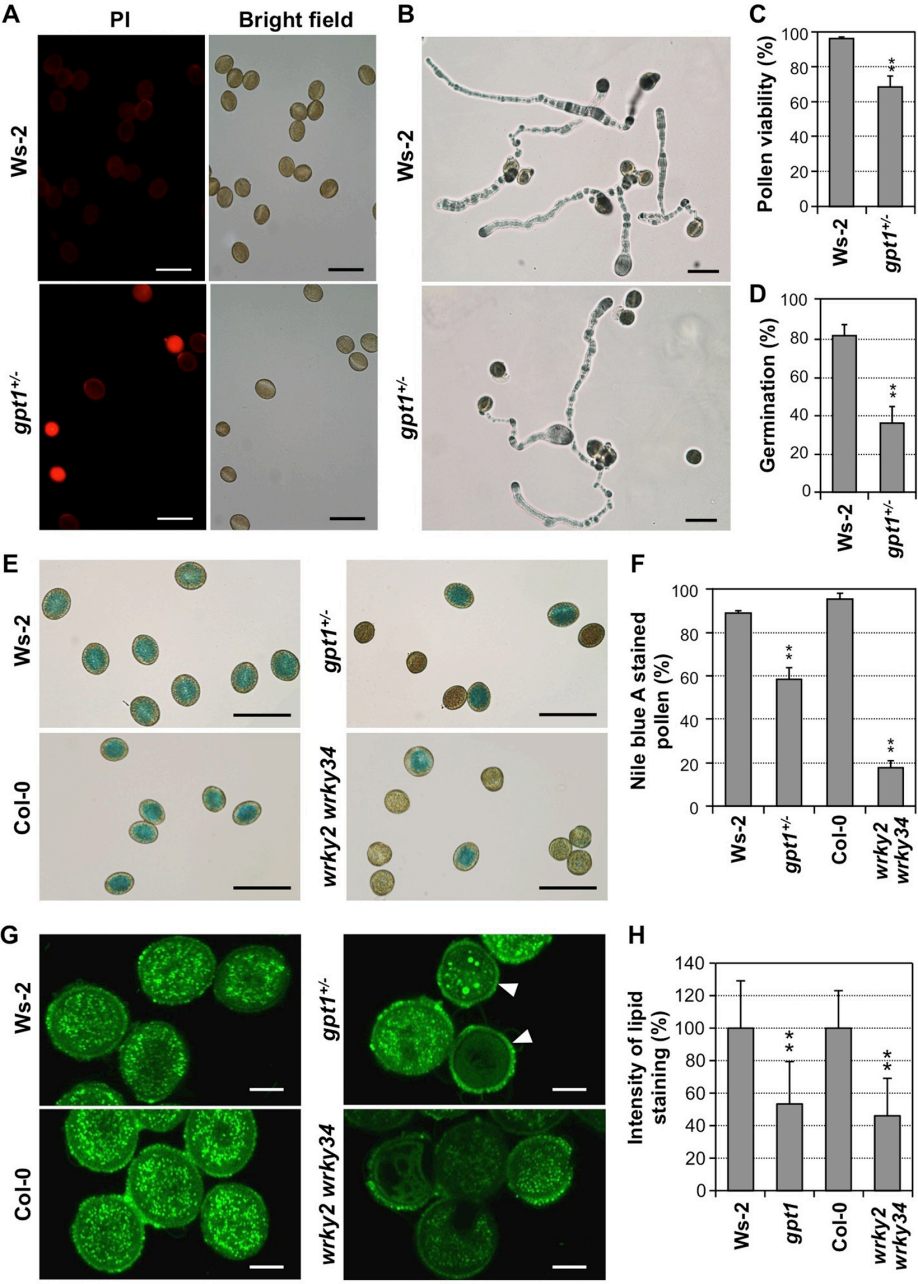
Phenotypic analysis of mature pollen from wild type (Ws-2 and Col-0), *gpt1*^*+/-*^ heterozygous, and *wrky2 wrky34* double mutant plants. (**A**) Reduced viability of pollen grains from *gpt1*^*+/-*^ plants in comparison to wild-type control (Ws-2) based on PI viability staining. Dead pollen grains show red fluorescence. (**B**) *In vitro* pollen germination of Ws-2 and *gpt1*^*+/-*^ plants. (**C**) Quantitation of pollen viability of Ws-2 and *gpt1*^*+/-*^ heterozygous mutant plants. (**D**) Quantitation of pollen germination rates of Ws-2 and *gpt1*^*+/-*^ heterozygous mutant. (**E**) Lipid staining of pollen from *gpt1*^*+/-*^, *wrky2 wrky34*, and their respective wild-type controls. Lipid-containing pollen is stained blue by Nile blue A. (**F**) Quantitation of Nile blue A-stainable pollen from *gpt1*^*+/-*^, *wrky2 wrky34*, and their respective wild-type control. (**G**) BODIPY 505/515 staining of lipid bodies in pollen of *gpt1*, *wrky2 wrky34*, and their respective wild-type controls. White arrows indicate smaller pollen grains, possibly of *gpt1* genotype. (**H**) Quantitation of fluorescence intensity in pollen of *gpt1*, *wrky2 wrky34*, and their respective wild-type controls. Intensity of fluorescence was quantified by ImageJ, and normalized to that in their respective wild-type controls, which was set as 100%. Only the smaller round pollen grains, which are likely of *gpt1* genotype, from *gpt1*^*+/-*^ plants were included in the quantification. In C, D, and F, at least 90 pollen grains were counted in each repeat. Error bars indicate SD (n = 3 in C, D, and F, and ≥ 10 in G). **P ≤ 0.01. Bar = 50 μm (A, B, and E) or 10 μm (G).

As the first step, we compared the pollen viability and germination of *wrky2 wrky34* double mutant and *gpt1*^*+/-*^ mutant side by side. Because *gpt1* homozygous mutant is embryo lethal, we used pollen from *gpt1*^*+/-*^ heterozygous mutant plants. The flowers and anthers of *gpt1*^*+/-*^ heterozygous mutant plants developed normally (S1 Fig). Propidium iodide (PI) staining, which gives dead pollen red fluorescence under the microscope [38], revealed a large proportion of dead pollen grains from *gpt1*^*+/-*^ heterozygous mutant plants. In contrast, pollen grains from wild-type Ws-2 control plants were mostly non-fluorescent, i.e. viable (Fig 2A). Quantitative analysis revealed 32 ± 6% (n = 5) of the pollen grains from *gpt1*^*+/-*^ heterozygous mutant plants were dead under our experimental conditions (Fig 2C). Using fluorescein diacetate (FDA), which stains viable pollen fluorescent green [35], we observed a similar percentage of non-viable pollen from *gpt1*^*+/-*^ plants (S2 Fig). Because FDA staining is not compatible with GPT1-eYFP fusion-rescued *gpt1* pollen (both have green fluorescence), PI staining was used throughout this study. We observed a higher percentage of dead pollen grains from *gpt1*^*+/-*^ plants than the previous report [36], probably due to different experimental conditions. Pollen grains from *gpt1*^*+/-*^ plants showed a significant reduction in *in vitro* germination rate, only 36 ± 8% (n = 3) relative to 82 ± 6% (n = 3) in Ws-2 wild type (Fig 2B and Fig 2D).

Pollen grains from *wrky2 wrky34* double mutant plants have similar phenotypes, with only a 37% viable rate and 28% germination rate [35]. However, these numbers are not directly comparable with those from *gpt1*^*+/-*^ plants because *gpt1*^*+/-*^ plants produce 50% wild-type pollen grains. Phenotypes of *gpt1* mutant pollen were attributed to defective fatty acid biosynthesis based on reduced lipid bodies in TEM images [36]. To determine whether the reduced viability of *wrky2 wrky34* pollen is associated with a decrease in lipid contents, we performed TEM analysis. As shown in S3 Fig, lipid bodies in *gpt1* pollen grains, similar to *wrky2 wrky34* pollen grains [35], were greatly reduced in comparison to the wild type. We also stained pollen with Nile blue A to detect the lipids. Pollen grains with sufficient lipids are stained blue. As shown in Fig 2E and 2F, approximately 58 ± 5% (n = 6) pollen grains from *gpt1*^*+/-*^ heterozygous plants and 18 ± 3% (n = 3) pollen grains from *wrky2 wrky34* double mutant plants were stained blue, which are much lower in comparison to their respectively wild-type controls, 89 ± 1% (n = 3) in Ws-2 and 95 ± 3% (n = 3) in Col-0. Because it is difficult to quantify the amount of fatty acid based on the intensity of Nile blue A staining, we used the percentage of positively stained pollen grains as a measure of the lipid accumulation in pollen as a population. Furthermore, we stained the pollen with BODIPY 505/515 to visualize the lipid bodies. As shown in Fig 2G, the number of lipid bodies in *gpt1* and *wrky2 wrky34* pollen was greatly reduced. Quantitation of fluorescence intensity indicated about 50% reduction in lipid body accumulation in the mutant pollen (Fig 2H). Based on these results, we can conclude that mutant pollen grains from both *gpt1*^*+/-*^ heterozygous mutant and *wrky2 wrky34* double mutant plants have a reduced level of storage lipids, which could lead to reduced pollen viability, germination, and transmission.

### Generation of a rescued *gpt1* system for functional analyses

Since pollen grains from *gpt1*^*+/-*^ heterozygous plants are a mixture of wild-type pollen and *gpt1* mutant pollen, it is difficult to 1) attribute a phenotype to pollen of a specific genotype, and 2) perform genetic analysis. To overcome these difficulties, we attempted to generate a rescued *gpt1* homozygous mutant system using fluorescent tagged GPT1, a strategy used in one of our previous studies [39]. A *GPT1* promoter-driven *GPT1-eYFP* fusion construct (*P*_*GPT1*_:*GPT1-eYFP*) was transformed into *gpt1*^*+/-*^ heterozygous plants. In T2 progenies, we successfully obtained *gpt1* homozygous plants with *P*_*GPT1*_:*GPT1-eYFP* single-insertion transgenes in homozygous state (genotype: *P*_*GPT1*_:*GPT1-eYFP gpt1*). They were then crossed with *gpt1*^*+/-*^ plants to obtain *P*_*GPT1*_:*GPT1-eYFP*^*+/-*^ *gpt1* plants. Successful rescue of *gpt1* mutant by *P*_*GPT1*_:*GPT1-eYFP* transgenes demonstrated that the transgene product is fully functional, which allowed us to 1) use the fusion protein to examine the spatiotemporal expression patterns of *GPT1*, and 2) identify the *gpt1* mutant pollen, which is non-fluorescent, from *P*_*GPT1*_:*GPT1-eYFP*^*+/-*^ *gpt1* plants. In addition, using a plastid marker construct *pt-rk CD3-999* [40], we demonstrated that GPT1-eYFP fusion co-localizes with a mCherry plastid marker (S4A Fig). This conclusion is consistent with the previous conclusion based on biochemical evidence [21].

**Fig 3:**
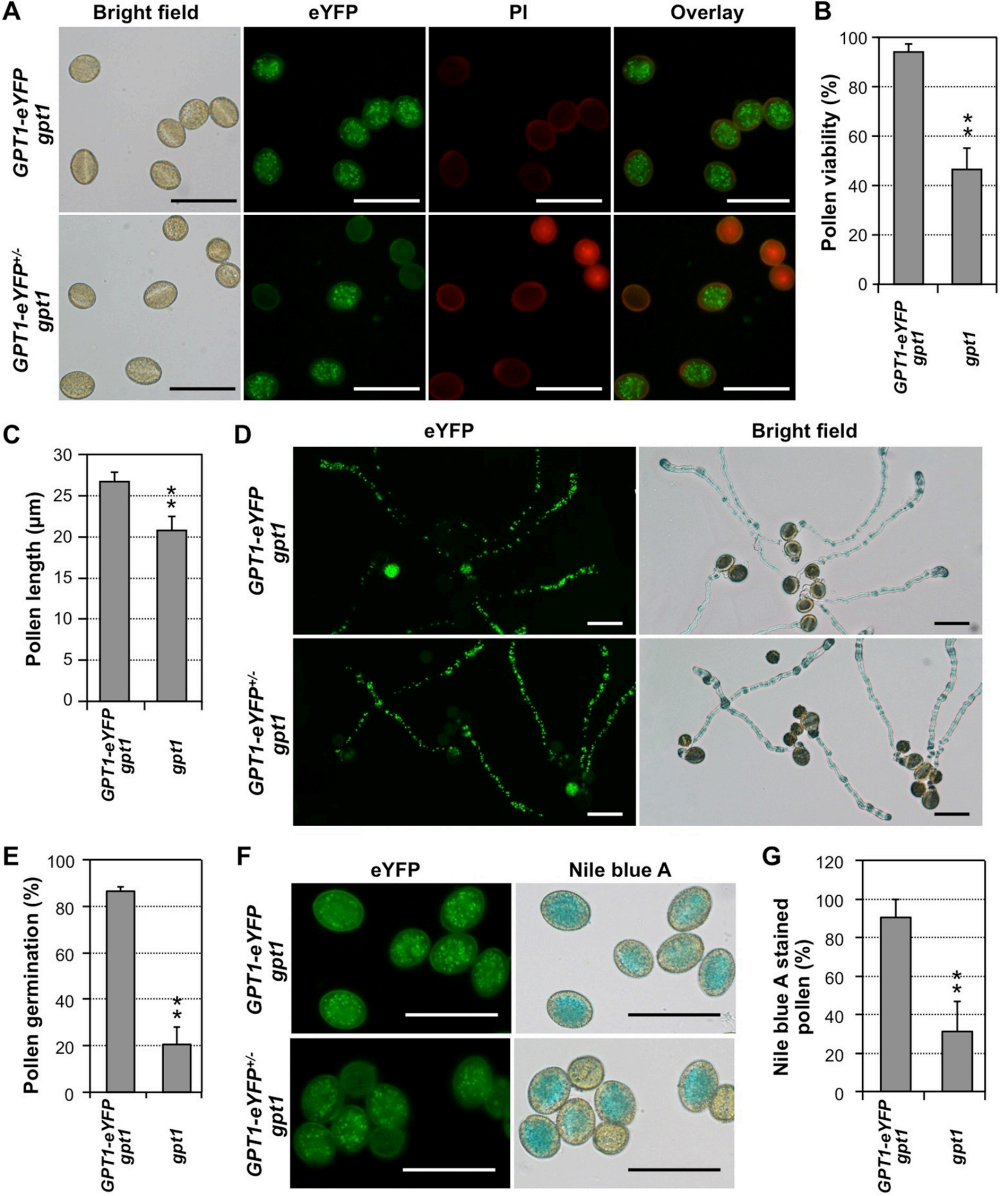
A GPT1-eYFP fusion-tagged *gpt1* system for pollen genotype identification during phenotypic analysis. (**A**) GPT1-eYFP fluorescence and PI staining of mature pollen grains from homozygous *P*_*GPT1*_: *GPT1-eYFP* rescued *gpt1* plants (upper) and heterozygous *P*_*GPT1*_:*GPT1-eYFP*^*+/-*^ rescued *gpt1* plants (lower). (**B**) Quantitation of pollen viability of *P*_*GPT1*_:*GPT1-eYFP gpt1* (fluorescent) and *gpt1* (non-fluorescent) pollen grains from *P*_*GPT1*_:*GPT1-eYFP*^*+/-*^ *gpt1* plants based on PI staining. (**C**) Sizes of *P*_*GPT1*_: *GPT1-eYFP gpt1* and *gpt1* pollen grains from *P*_*GPT1*_:*GPT1-eYFP*^*+/-*^ *gpt1* plants. (**D**) *In vitro* germination of pollen from *P*_*GPT1*_: *GPT1-eYFP gpt1* (upper) and *P*_*GPT1*_:*GPT1-eYFP*^*+/-*^ *gpt1* (lower) plants. (**E**) Quantitation of pollen germination rates of *P*_*GPT1*_:*GPT1-eYFP gpt1* and *gpt1* pollen. (**F**) GPT1-eYFP fluorescence and Nile blue A lipid staining of pollen from *P*_*GPT1*_:*GPT1-eYFP gpt1* (upper) and *P*_*GPT1*_:*GPT1-eYFP*^*+/-*^ *gpt1* (lower) plants. (**G**) Quantitation of lipid-positive pollen grains with either *GPT1-eYFP gpt1* or *gpt1* genotypes. In the quantitative assays (B, C, E, and G), at least 80 pollen grains were counted in each repeat. Three independent *P*_*GPT1*_:*GPT1-eYFP* transgenic lines in *gpt1* background were analyzed with similar results. Data from one of the lines are shown. Error bars indicate SD (n ≥ 3). **P ≤ 0.01. Bar = 50 μm.

Pollen viability assay revealed that *P*_*GPT1*_:*GPT1-eYFP gpt1* plants had wild-type phenotype (Fig 3A, upper panels). All pollen grains from *P*_*GPT1*_:*GPT1-eYFP gpt1* plants had eYFP signal, showed normal morphology, and were PI-unstainable. Half of the pollen grains from *P*_*GPT1*_:*GPT1-eYFP*^*+/-*^ *gpt1* plants did not have eYFP signal (Fig 3A, lower panels). They were *gpt1* mutant pollen grains. A large percentage of them were PI-stainable nonviable pollen (Fig 3A, lower panels). In contrast, pollen grains with eYFP signal (~50%) from *P*_*GPT1*_:*GPT1-eYFP*^*+/-*^ *gpt1* plants, which were complemented *gpt1* pollen (genotype: *gpt1 P*_*GPT1*_:*GPT1-eYFP*), showed wild-type phenotype. Fig 3B showed that only 47 ± 8% (n = 4) of *gpt1* pollen grains was viable, which was significantly lower than the value of *P*_*GPT1*_:*GPT1-eYFP gpt1* pollen, 94 ± 4% (n = 4). Such a decrease was consistent with the reduction of pollen viability from ~96% in the wild-type plants to ~68% in the *gpt1*^*+/-*^ heterozygous plants (Fig 2C). The viable *gpt1* mutant pollen grains also suggest the presence of other transporter(s), such as GPT2 and TPT, and/or pathway(s) that can compensate the loss of *GPT1* and synthesize sufficient amount of lipids.

We also noticed that all non-fluorescent pollen grains (genotype: *gpt1*) were smaller and more rounded in shape, while the fluorescent GPT1-eYFP*-*rescued pollen grains (genotype: *P*_*GPT1*_:*GPT1-eYFP gpt1*) were bigger and oval shaped (Fig 3A, lower panels). The length of the *gpt1* pollen grains was 20.8 ± 1.6 μm (n = 156), significantly smaller than the value of 26.6 ± 1.2 μm (n = 185) for the rescued fluorescent pollen grains (Fig 3C). Although this phenotype could be observed in the mixed pollen grains from *gpt1*^*+/-*^ plants (Fig 2A), the genotype of these smaller pollen grains was not clear. *In vitro* pollen germination assay showed a major reduction in *gpt1* pollen germination rate, from 86 ± 2% (n = 5) for *P*_*GPT1*_:*GPT1-eYFP gpt1* pollen to 21 ± 7% (n = 5) for *gpt1* pollen (Fig 3D and 3E). Pollen from *P*_*GPT1*_:*GPT1-eYFP gpt1* plants had a germination rate (82 ± 1%, n = 3) similar to wild type (82 ± 6%, n = 3) (Fig 2B and Fig 3D, upper panels).

Nile blue A staining revealed that pollen from *P*_*GPT1*_:*GPT1-eYFP gpt1* plants all was stained blue and had green fluorescence (Fig 3F, upper panels). Among the pollen grains from *P*_*GPT1*_:*GPT1-eYFP*^*+/-*^ *gpt1* plants, only 31 ± 15% (n = 5) of the non-fluorescent pollen grains (*gpt1* mutant pollen) could be stained blue, while 90 ± 9% (n = 5) of the fluorescent pollen grains (*P*_*GPT1*_:*GPT1-eYFP gpt1* rescued pollen) were stained blue (Fig 3F, lower panels, and Fig 3G). In summary, using a rescued *gpt1* homozygous mutant system with *GPT1-eYFP* fusion transgene, we were able to quantitatively define the viability, size, germination rate, and lipid accumulation in *gpt1* pollen, which would otherwise be impossible to identify specifically. The *gpt1* pollen grains are smaller with reduced lipid accumulation, and have reduced viability and germination rate, which is consistent with its reduced transmission rate of 20% (n = 526) based on backcross using pollen from *gpt1*^*+/-*^ plants.

### Temporal expression of *GPT1* in pollen is dependent on WRKY2 and WRKY34 transcription factors and correlates with the accumulation of lipid bodies

**Fig 4:**
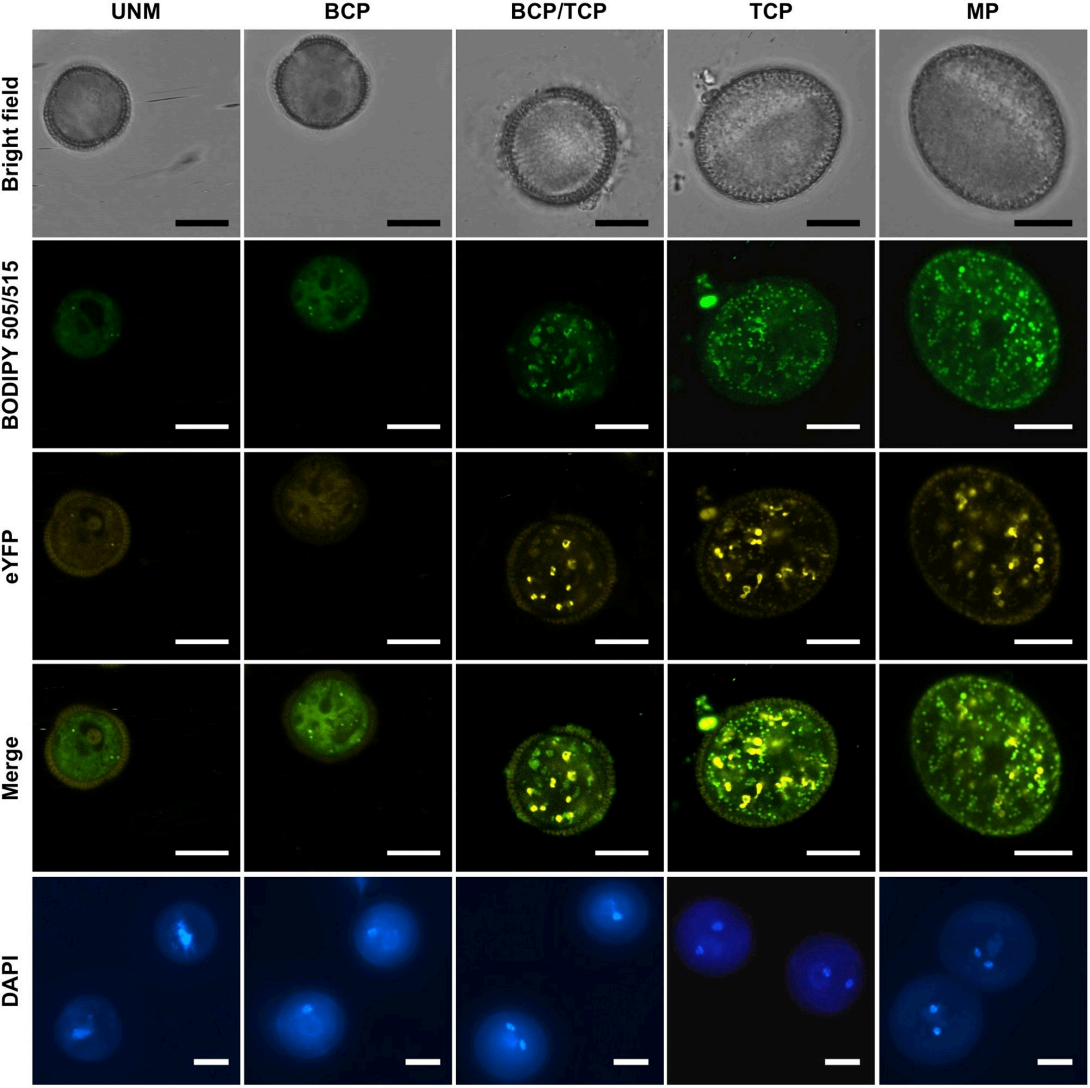
Expression of GPT1-eYFP fusion correlates with the accumulation of lipid bodies during pollen development. Pollen grains from *P*_*GPT1*_:*GPT1-eYFP gpt1* plants at different development stages were imaged. First row: bright field images to show pollen morphology; second row: BODIPY 505/515 staining to show the accumulation of lipid bodies at the late stages of pollen development; third row: eYFP images to show the expression of GPT1-eYFP; fourth row: merged images of BODIPY 505/515 staining of lipid bodies and eYFP fluorescence of GPT1-eYFP fusion; and fifth row: DAPI staining of pollen grains from the same anthers for determination of pollen nuclear stage. UNM, uninucleate microspore; BCP, bicellular pollen; TCP, tricellular pollen; and MP, mature pollen. Three independent *P*_*GPT1*_:*GPT1-eYFP* transgenic lines in *gpt1* background were analyzed with similar results. Images from one of the lines are shown. Bar = 10 μm.

Using the fully complemented *P*_*GPT1*_:*GPT1-eYFP gpt1* plants, we analyzed the expression pattern of *GPT1* reporter during pollen development. As shown in Fig 4, GPT1-eYFP fluorescence was not visible in uninucleate microspores (UNM) and early bicellular pollen (BCP). It became detectable at late BCP stage and early tricellular pollen (TCP), peaked at TCP stage, and stayed high in mature pollen (MP). GPT1-eYFP fusion protein appeared as small speckles in pollen, consistent with its localization on plastids (S4 Fig). The accumulation of GPT1-eYFP is preceded by the appearance of WRKY2 and WRKY34 proteins in the vegetative nucleus [35]. As shown in the Fig 4B to 4Q of Guan et al paper [35], both WRKY2 and WRKY34 proteins reached their peak levels at BCP stage, which is consistent with a role of these two WRKYs in regulating *GPT1* expression. To visualize the accumulation of lipid bodies during pollen development, we used BODIPY 505/515 staining. As shown in Fig 4, no or very few lipid bodies were visible in pollen at UNM and BCP stages. At later stages, there was an accumulation of lipid bodies, concurrently with the increase in GPT1-eYFP protein. Together with the loss-of-function genetic evidence, we can conclude that GPT1 plays a key role in lipid body accumulation during pollen maturation.

**Fig 5:**
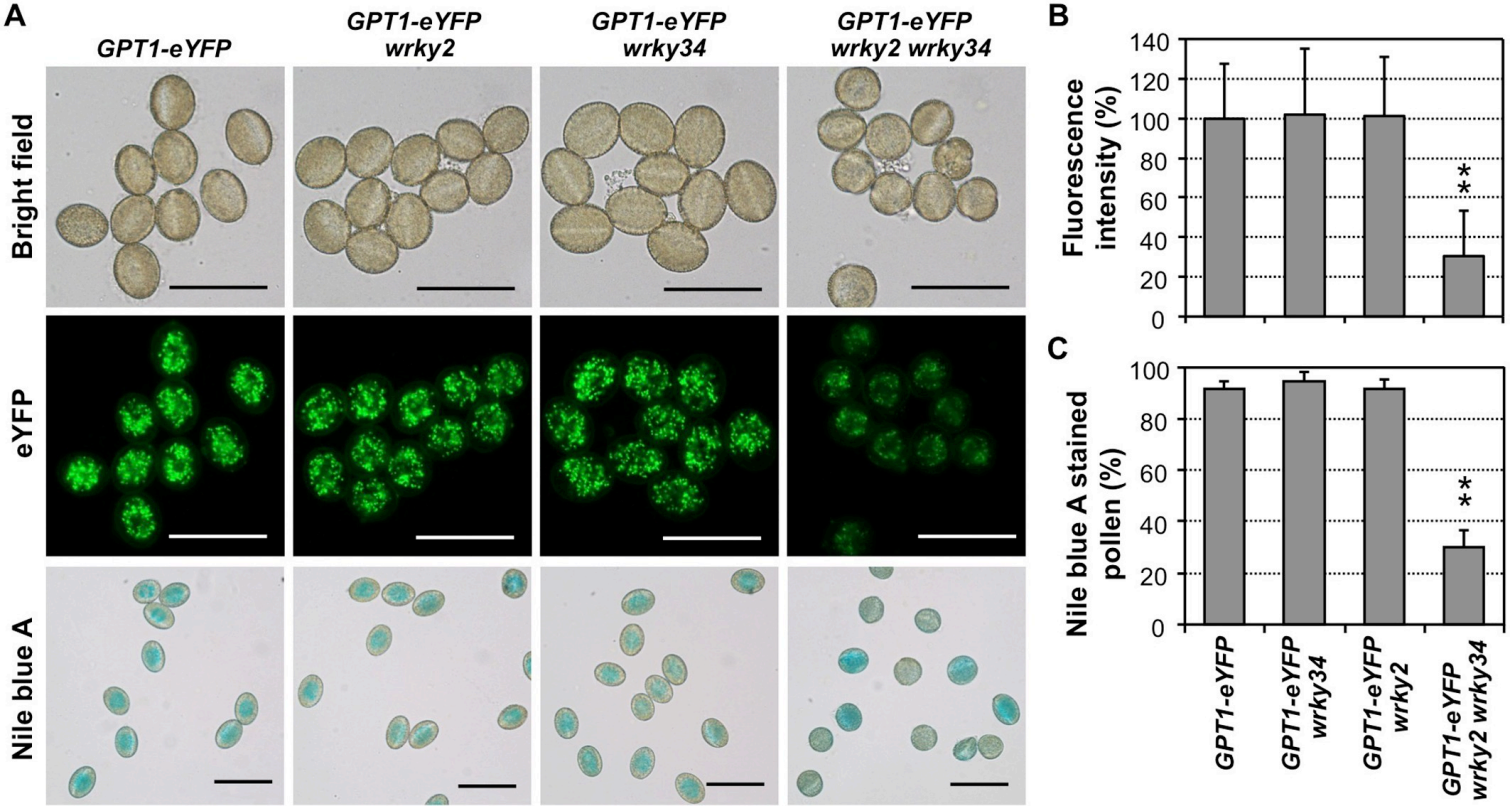
Loss of function of both *WRKY2* and *WRKY34* compromises *GPT1* expression and storage lipid biosynthesis in pollen. (**A**) Bright-field images (top panels) and eYFP images of the same fields (middle panels) of pollen from *P*_*GPT1*_:*GPT1-eYFP*, *P*_*GPT1*_:*GPT1-eYFP wrky2*, *P*_*GPT1*_:*GPT1-eYFP wrky34*, and *P*_*GPT1*_:*GPT1-eYFP wrky2 wrky34* plants. Lipid accumulation in pollen from these plants was detected by Nile blue A lipid staining (bottom panels). (**B**) Quantitation of fluorescence intensity in pollen grains of different genotypes. (**C**) Quantitation of Nile blue A-positive pollen in different genotypes. At least 130 pollen grains were counted in each repeat. Two independent *P*_*GPT1*_:*GPT1-eYFP* transgenic lines in wild-type and *wrky* single/double mutant backgrounds were analyzed with similar results. Results from one of them are shown. Error bars indicate SD (n = 3). **P ≤ 0.01. Bar = 50 μm.

To determine whether *GPT1* expression during pollen development is regulated by WRKY2 and WRKY34, we transformed *wrky2 wrky34* double mutant plants with *P*_*GPT1*_:*GPT1-eYFP* construct. T2 homozygous plants with single transgene insertion were crossed with Col-0 to generate *P*_*GPT1*_:*GPT1-eYFP*, *P*_*GPT1*_:*GPT1-eYFP wrky2*, *P*_*GPT1*_:*GPT1-eYFP wrky34*, and *P*_*GPT1*_:*GPT1-eYFP wrky2 wrky34* plants. We then compared the GPT1-eYFP signal in pollen grains from these four genotypes with the same transgene allele. As shown in Fig 5A, GPT1-eYFP signal in single *wrky2* and single *wrky34* mutant background was the same as that in the wild-type background based on quantification of fluorescence intensity (Fig 5B). However, the GPT1-eYFP signal in the *wrky2 wrky34* double mutant background was only about 30% of the wild type (Fig 5A and 5B). This result is consistent with the lower expression of native *GPT1* in the anthers of *wrky2 wrky34* double mutant plants (Fig 1), providing further support that *GPT1-eYFP* expression is dependent on the functional *WRKY2* and *WRKY34*.

We next stained pollen grains from these four genotypes with Nile blue A to determine whether reduced expression of *GPT1-eYFP* in *wrky2 wrky34* double mutant pollen would result in a reduction in lipid accumulation. As shown in Fig 5A (lower panels), pollen grains in *wrky34* or *wrky2* single mutant background accumulated lipids at a level similar to the wild type. In contrast, the majority of the pollen grains in *wrky2 wrky34* double mutant background could not be stained. Quantitative analyses indicated that only 30 ± 6% (n = 3) of the pollen grains from *P*_*GPT1*_:*GPT1-eYFP wrky2 wrky34* plants was stained blue using Nile blue A assay, which was significantly lower than those from *P*_*GPT1*_:*GPT1-eYFP*, *P*_*GPT1*_:*GPT1-eYFP wrky34*, and *P*_*GPT1*_:*GPT1-eYFP wrky2* plants with percentages of 92 ± 2% (n = 3), 95 ± 3% (n = 3), and 92 ± 3% (n = 3), respectively (Fig 5C). BODIPY 505/515 staining further confirmed the compromised accumulation of lipid bodies in *wrky2 wrky34* double mutant pollen (S5 Fig).

### Lethality of *gpt1* and *wrky2 wrky34* pollen occurs at the late stages of pollen development, which correlates with the compromised lipid body accumulation

Loss of function of *GPT1* or *WRKY2/WRKY34* leads to compromised accumulation of lipid bodies at the late stages (TCP and MP) of pollen development and reduced pollen viability. To determine when the cell death occurred in *gpt1* and *wrky2 wrky34* pollen, we first performed DAPI staining of pollen nuclei at different pollen developmental stages. As shown in S6 Fig, *gpt1* mutant pollen was not distinguishable morphologically at the early stages (UNM to early TCP), despite their smaller sized at maturity (Figs 2 and 3). In addition, all pollen grains progressed to TCP stage, suggesting that the loss of *GPT1* has minimal effects on pollen development before maturation, and pollen collapse/death is a late event during maturation process. Consistent with this, PI viability staining revealed pollen death only at TCP and MP stages (S8 Fig). Similar results were observed in *wrky2 wrky34* pollen (S7 and S8 Figs).

In *Arabidopsis*, it is known that mature pollen contains mostly lipid bodies, although starch is present in the vegetative cell at the early stages of pollen development [12, 13]. To determine whether *gpt1* or *wrky2 wrky34* mutation has an impact on starch accumulation in pollen, we performed Lugol’s iodine staining. As shown in S9 and S10 Figs, no change in starch accumulation was observed in *gpt1* or *wrky2 wrky34* mutant pollen. Based on the biochemical function of GPT1 protein in transporting Glc6P into plastids, it is more likely that the mutation of *GPT1* results in the loss/reduction of carbon source and/or NADPH needed for fatty acid biosynthesis in plastids, and subsequently lipid body accumulation during pollen maturation, which then leads to the reduction of pollen viability.

### Pollen-specific overexpression of *GPT1* rescues the pollen phenotypes of *wrky2 wrky34* double mutant

**Fig 6:**
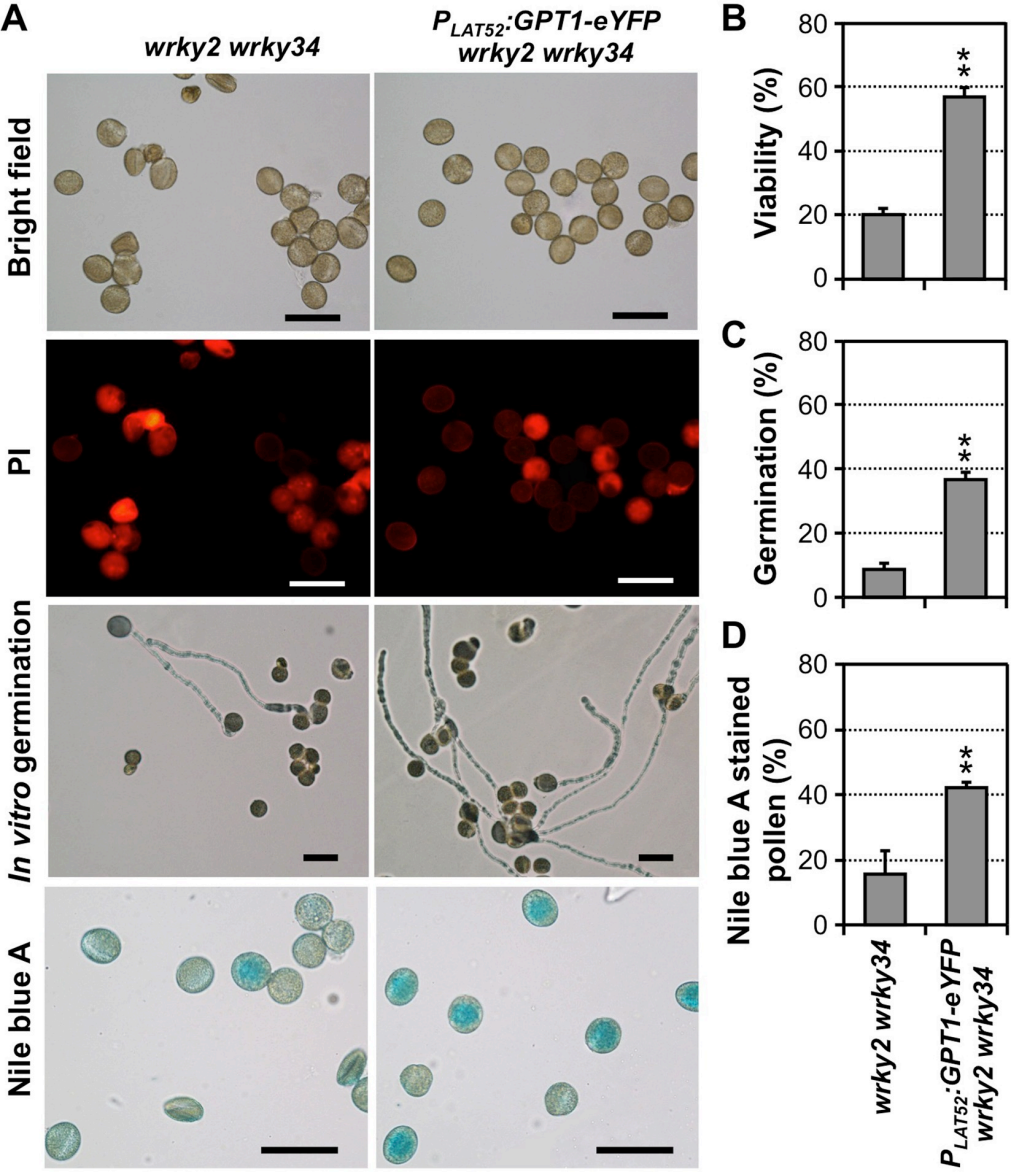
Pollen-specific overexpression of GPT1-eYFP rescues the pollen defects of *wrky2 wrky34* double mutant plants. (**A**) Viability (as indicated by PI staining), *in vitro* germination, and lipid accumulation (as indicated by Nile blue A staining) of pollen grains from *wrky2 wrky34* and *P*_*LAT52*_:*GPT1-eYFP wrky2 wrky34* plants. **(B)** Quantitation of pollen viability of *wrky2 wrky34* and *P*_*LAT52*_:*GPT1-eYFP wrky2 wrky34*. (**C**) *In vitro* germination rates of pollen from *wrky2 wrky34* and *P*_*LAT52*_:*GPT1-eYFP wrky2 wrky34* plants. (**D**) Quantitation of Nile blue A-stainable pollen with either *wrky2 wrky34* or *P*_*LAT52*_:*GPT1-eYFP wrky2 wrky34* genotypes. At least 150 pollen grains were counted in each repeat. Three independent *P*_*LAT52*_:*GPT1-eYFP* transgenic lines in *wrky2 wrky34* background were analyzed and all gave similar results. Results from one of the three lines are shown. Error bars indicate SD (n = 3). **P ≤ 0.01. Bar = 50 μm.

To genetically test whether *GPT1* functions downstream of *WRKY2* and *WRKY34* in regulating pollen storage lipid accumulation, we performed epistatic analysis by overexpressing *GPT1* gene in *wrky2 wrky34* background. We transformed *wrky2 wrky34* double mutant with GPT1-eYFP fusion driven by *LAT52*, a strong pollen-specific promoter [41]. T3 homozygous lines (genotype: *P*_*LAT52*_:*GPT1-eYFP wrky2 wrky34*) were selected for experiments. Pollen viability assay revealed that, while the majority of *wrky2 wrky34* pollen grains showed PI fluorescence, a much smaller percentage of *P*_*LAT52*_:*GPT1-eYFP wrky2 wrky34* pollen had red fluorescence (Fig 6A). Quantitative analyses showed that *P*_*LAT52*_:*GPT1-eYFP wrky2 wrky34* plants had a higher percentage of live pollen (57 ± 11%, n = 3) than *wrky2 wrky34* double mutant plants (20 ± 3%, n = 3) (Fig 6B).

In pollen germination assay, 37 ± 2% (n = 3) of the pollen grains from *P*_*LAT52*_:*GPT1-eYFP wrky2 wrky34* plants germinated, a percentage 4-times as high as the germination rate of *wrky2 wrky34* pollen (9 ± 2%, n = 3) under the same conditions (Fig 6A and 6C). Furthermore, we stained the pollen from *wrky2 wrky34* and *P*_*LAT52*_:*GPT1-eYFP wrky2 wrky34* with Nile blue A. As shown in Fig 6A and 6D, approximately 42 ± 2% (n = 5) of the pollen grains from *P*_*LAT52*_:*GPT1-eYFP wrky2 wrky34* plants could be stained blue, i.e. with normal accumulation of storage lipids. In contrast, only 16 ± 7% (n = 5) of the pollen grains from *wrky2 wrky34* double mutant plants were stained blue. BODIPY 505/515 staining further demonstrated the restoration of lipid body accumulation in *wrky2 wrky34* pollen with pollen-specific overexpression of *GPT1* (S11 Fig). These data are consistent with the higher pollen viability and pollen germination of *P*_*LAT52*_:*GPT1-eYFP wrky2 wrky34* plants. The successful rescue of *wrky2 wrky34* double mutant pollen phenotypes by pollen-specific overexpression of *GPT1* demonstrates that *GPT1* is a major target gene of WRKY2 and WRKY34 transcription factors in the regulation of lipid body biogenesis during pollen maturation.

### The W-boxes in *GPT1* promoter are required for the high-level *GPT1* expression in pollen

**Fig 7:**
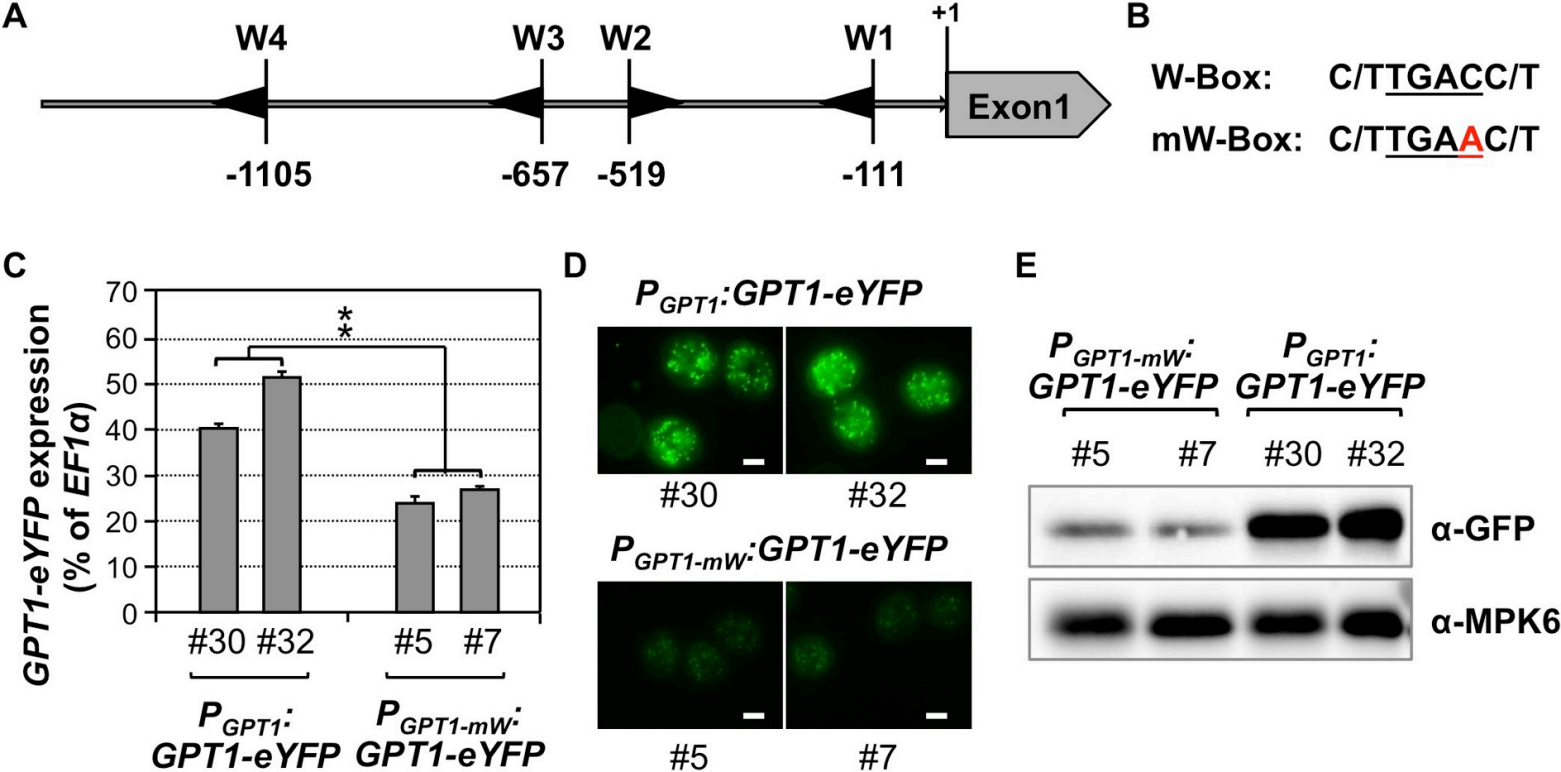
W-boxes in *GPT1* promoter are required for high expression of GPT1-eYFP reporter. (**A**) Diagrams showing the W-boxes in the 1.2-kb region upstream of the translation initiation site of *GPT1*. Black arrows represent W-boxes. (**B**) Sequence of wild-type W-box and mutated W-box. (**C**) Gene expression of *GPT1-eYFP* driven by native *GPT1* promoter with wild-type or mutated W-boxes. *GPT1-eYFP* transcript levels were quantitated by real-time qPCR. *EF1α* was used as an internal control. Values are means ± SD, n = 3. **P ≤ 0.01. (**D**) GPT1-eYFP fluorescence in pollen from *P*_*GPT1*_:*GPT1-eYFP*^*+/-*^ and *P*_*GPT1-mW*_: *GPT1-eYFP*^*+/-*^ plants. (**E**) Immunoblot analysis of GPT1-eYFP protein levels in pollen of *P*_*GPT1*_:*GPT1-eYFP*^*+/-*^ and *P*_*GPT1-mW*_: *GPT1-eYFP*^*+/-*^ plants with anti-GFP antibody (upper panels). The blot was striped and reprobed with anti-MPK6 to show equal protein loading (lower panels). Multiple T1 lines with similar phenotype were obtained. Results from two representative lines are shown. Bar = 10 μm.

W-box is the cis-element known to be the binding site of WRKY transcription factors [42]. *GPT1* promoter contains four copies of W-boxes within the 1.2 kb region (Fig 7A). To test whether these four W-boxes in the *GPT1* promoter are important to *GPT1* expression, we mutated the core sequence (TGAC) of all four W-boxes to TGAA (named mutated W-box, or mW) and compared its activity with the wild-type *GPT1* promoter. *GPT1-eYFP* fusion driven by wild-type promoter (*P*_*GPT1*_:*GPT1-eYFP*) and mutated promoter (*P*_*GPT1-mW*_:*GPT1-eYFP*) were introduced into wild-type plants. Experiments in Fig 7 compared two representative lines of wild-type promoter and mW promoter transgenes. Quantitative RT-PCR revealed an approximately two-fold decrease in *GPT1-eYFP* transcripts when the W boxes in the *GPT1* promoter were mutated (Fig 7C). The decrease in transcript was accompanied by a reduced GPT1-eYFP fluorescent signal (Fig 7D) and reduced protein level (Fig 7E) in the *P*_*GPT1-mW*_:*GPT1-eYFP* lines based on microscopy observation and western blot analysis, respectively. Yeast one-hybrid assay further confirmed the binding of WRKY34 transcription factor to *GPT1* promoter (S12 Fig). Furthermore, mutation of the W-boxes in *GPT1* promoter abolished this interaction. Compromised promoter activity after the mutation of W-boxes in *GPT1* promoter provides another line of evidence that the W-boxes in the *GPT1* promoter are important to the activation of *GPT1* expression by WRKY2 and WRKY34 during pollen development.

### MPK3/MPK6 cascade plays an important role in lipid biosynthesis by controlling *GPT1* expression during pollen development

**Fig 8:**
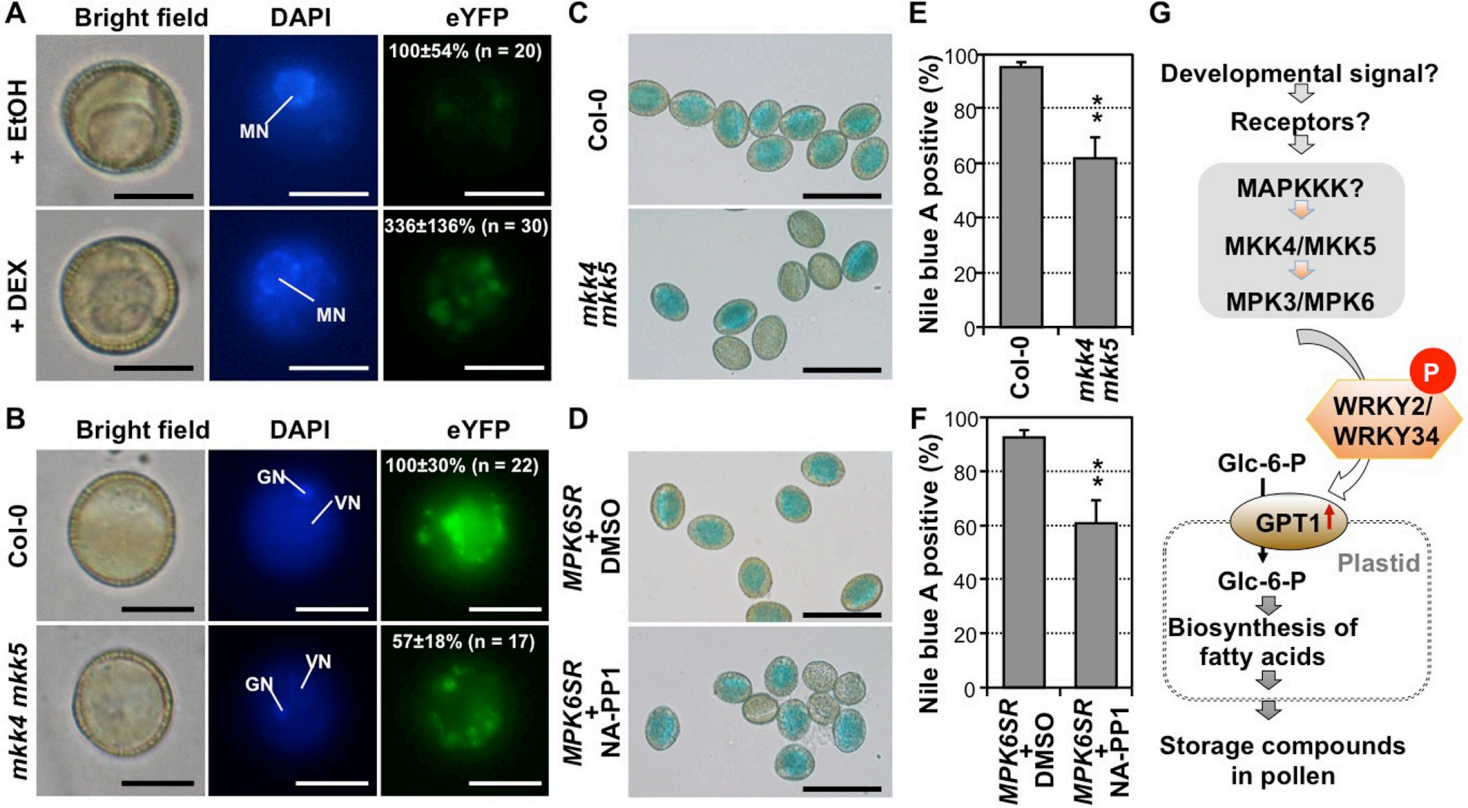
Gain-of-function activation of MPK3/MPK6 promotes ectopic overexpression of *GPT1* and loss-of-function of *MKK4/MKK5* leads to a reduced *GPT1* expression and lipid accumulation. (**A**) Bright-field, DAPI-staining, and GPT1-eYFP fluorescence images of pollen from *P*_*GPT1*_:*GPT1-eYFP DD* plants treated with either ethanol control (+EtOH) or DEX. The intensity of eYFP fluorescence was quantified, and normalized to that in the +EtOH control, which was set as 100%. The difference in the intensity of eYFP fluorescence between DEX treated and EtOH control was very significant (P<0.01). Five independent *P*_*GPT1*_:*GPT1-eYFP* transgenic lines in *DD* background were analyzed and all gave similar results. Results from one of the five lines are shown. MN, microspore nucleus. Bar = 10 μm. (**B**) Bright-field, DAPI-staining, and GPT1-eYFP fluorescence images of pollen from *P*_*GPT1*_:*GPT1-eYFP* and *P*_*GPT1*_:*GPT1-eYFP mkk4 mkk5* plants. The intensity of eYFP fluorescence was quantified, and normalized to that in the Col-0 background, which was set as 100%. The difference in eYFP fluorescence intensity between *mkk4 mkk5* double mutant and Col-0 control was very significant (P<0.01). Two independent *P*_*GPT1*_:*GPT1-eYFP* transgenic lines in both wild-type and *mkk4 mkk5* backgrounds were analyzed with similar results. One of them is shown. VN, vegetative nucleus. GN, generative nucleus. Bar = 10 μm. (**C**) Nile blue A lipid staining of pollen from Col-0 and *mkk4 mkk5* plants. (**D**) Nile blue A lipid staining of pollen from chemical genetically rescued *MPK6SR* plants treated with either DMSO solvent or NA-PP1. (**E**) Quantitation of Nile blue A-stainable pollen from Col-0 or *mkk4 mkk5* plants. (**F**) Quantitation of Nile blue A-positive pollen from DMSO- and NA-PP1 treated *MPK6SR* plants. At least 100 pollen grains were counted in each repeat. Error bars indicate SD (n = 3). **P ≤ 0.01. Bar = 50 μm. (**G**) Working model depicts the function of plastid-localized GPT1 downstream of MKK4/MKK5—MPK3/MPK6—WRKY2/WRKY34 signaling pathway in controlling lipid body accumulation during pollen maturation.

We then examined the involvement of MPK3/MPK6, and their upstream MKK4/MKK5, in *GPT1* expression and lipid accumulation. Previously, we demonstrated that MPK3/MPK6 phosphorylate WRKY34, and possibly WRKY2, and are involved in pollen development [35]. We first transformed the native promoter-driven *GPT1-eYFP* construct into the conditional gain-of-function *GVG-NtMEK2*^*DD*^ (abbreviated as *DD*) background to generate *P*_*GPT1*_:*GPT1-eYFP DD* plants, and then examined the GPT1-eYFP signal in pollen after dexamethasone (DEX) treatment. Induction of the constitutively active DD protein leads to the activation of downstream endogenous MPK3/MPK6 [34, 43, 44]. As shown in Fig 8A, GPT1-eYFP signal in pollen from *P*_*GPT1*_: *GPT1-eYFP DD* plants treated with DEX was more than three times stronger than that in pollen from the same plants treated with ethanol solvent control, demonstrating that gain-of-function activation of MPK3/MPK6 is sufficient to promote the ectopic expression of *GPT1*. In this experiment, we examined pollen grains at the late UNM stage when the endogenous GPT1 signal has not been turned on yet and *GPT1* expression is very low (Fig 4). At later pollen development stages, the gain-of-function phenotype was not very obvious because of the high *GPT1* gene activation by the endogenous signaling pathway.

In a loss of function experiment, we examined *GPT1* expression and pollen lipid accumulation in the newly generated *mkk4 mkk5* double TILLING mutant [45]. MKK4 and MKK5 play redundant function upstream of MPK3 and MPK6 in various processes [31, 46]. We first transformed *P*_*GPT1*_:*GPT1-eYFP* construct into *mkk4 mkk5* double mutant background, selected lines with single transgene insertions, and then crossed the transgene alleles into the wild-type background. We found that pollen grains from *P*_*GPT1*_:*GPT1-eYFP mkk4 mkk5* plants (Fig 8B) had much weaker (~50%) GPT1-eYFP signal than that from *P*_*GPT1*_:*GPT1-eYFP* plants. Furthermore, Nile blue A staining revealed that less pollen grains were stained blue in *mkk4 mkk5* double mutant background, 62 ± 8% (n = 6) in comparison to 95 ± 2% (n = 6) in Col-0 wild type (Fig 8C and 8E). BODIPY 505/515 staining revealed that the accumulation of lipid bodies was significantly reduced in *mkk4 mkk5* double mutant pollen, but not *mkk4* or *mkk5* single mutant pollen, demonstrating a redundant function of *MKK4* and *MKK5* in the process (S13 Fig).

We further examined storage lipid accumulation in loss-of-function *mpk3 mpk6* pollen. A chemical-genetically rescued *mpk3 mpk6* double mutant system [47] was utilized. Because homozygous *mpk3 mpk6* double mutant is embryo lethal, we used a chemical-sensitized version of MPK6, *MPK6*^*YG*^, to rescue the double *mpk3 mpk6* mutant, and the resulting plants were named *MPK6SR* plants (genotype: *mpk3 mpk6 P*_*MPK6*_:*MPK6*^*YG*^). The kinase activity of MPK6^YG^ can be specifically inhibited by 4-amino-1-tert-butyl-3-(1’-naphthyl)pyrazolo[3,4-d]pyrimidine (NA-PP1), a derivative of PP1 kinase inhibitor with a bulky side chain that cannot enter the ATP binding pocket of other kinases [48]. As shown in Fig 8D and 8F, pollen grains from *MPK6SR* plants treated with NA-PP1 had reduced fatty acid. Only 61 ± 9% (n = 5) of the pollen grains were stained blue in Nile blue A assay, while this value was 93 ± 3% (n = 5) for DMSO-solvent control treated *MPK6SR* plants. Furthermore, BODIPY 505/515 staining revealed compromised lipid body accumulation in *MPK6SR* plants treated with NA-PP1, but not DMSO control (S14 Fig). Together, these experiments provide loss-of-function evidence to support the role of MPK3/MPK6 in storage lipid accumulation during pollen maturation.

## Discussion

Development of male gametophyte from a uninucleate microspore to a mature pollen grain involves precise control of gene expression, although the signaling pathways are largely unexplored. Previously, we reported that WRKY2 and WRKY34 function downstream of MPK3/MPK6 in pollen development [35]. In this study, we identified *GPT1* as an important target gene of WRKY2/WRKY34 based on molecular, cellular, and genetic analyses. *GPT1* expression in pollen is temporal-specific, and reaches its highest level during TCP and MP stages (Fig 4). This is consistent with its biological function in the biosynthesis and accumulation of storage lipids during pollen maturation process in *Arabidopsis* (Figs 2 and 3). The expression of *GPT1* is preceded by the induction and MPK3/MPK6-mediated phosphorylation of WRKY2/WRKY34 during pollen development (Fig 4) [35]. In addition, loss of function of either WRKY2/WRKY34 or MKK4/MKK5-MPK3/MPK6 module compromises the *GPT1* expression, lipid accumulation, and pollen functions (Figs 2, 5, 7, and 8; S5, S13 and S14 Figs), and gain-of-function activation of MPK3/MPK6 induces ectopic expression of GPT1 (Fig 8A). The fact that overexpression of *GPT1* using a pollen-specific promoter could rescue the pollen phenotype of *wrky2 wrky34* double mutant strongly supports that *GPT1* is a major target gene of WRKY2/WRKY34 (Fig 6, and S11 Fig).

### Regulation of metabolic activity in plastids by a cytoplasmic/nuclear signaling pathway

Lipid bodies and/or starch granules stored in the vegetative cytoplasm of the mature pollen provide carbon source material and energy to support the rapid pollen tube growth [4, 49, 50]. The lack of storage compounds as a result of either developmental defect or environmental stress greatly limits plant reproduction. It is known that accumulation of storage compounds happens at the late stage of the pollen development in all plants. However, the signaling pathway that controls this process was unclear. The identification of a MAPK signaling pathway, its downstream WRKY transcription factors, and *GPT1*, a key target gene of WRKY2/WRKY34 transcription factors, greatly advances our understanding of this process. GPT1 is directly involved in the lipid biosynthesis by transporting Glc6P into the plastids of heterotrophic pollen where Glc6P can be converted to acetyl-CoA and used to generate reducing equivalent for fatty acid biosynthesis.

Using a fully functional eYFP fusion reporter, we demonstrated that GPT1 protein starts to accumulate in BCP/TCP (Fig 4), which is consistent with the findings that lipid bodies accumulate after the first mitosis and rapidly fill up the cytoplasm of the vegetative cell [4, 8, 12]. The identification of a cytoplasmic/nuclear signaling pathway that regulates the metabolic activities in plastids (Fig 8G) greatly advanced our understanding of the coordination/regulation of plant metabolism in different cellular compartments. We speculate that the regulation of pollen storage compounds involves developmental signal(s) sensed by pollen surface receptor(s), which then activate the MPK3/MPK6 cascade. The phosphorylation of WRKY transcription factors by MPK3/MPK6 leads to the activation of *GPT1* gene expression (Fig 8G). This, together with other metabolic enzymes, gives the undifferentiated plastids the capacity to synthesize fatty acids, therefore, specifies the function of plastids in pollen at late development stages.

### GPT1, a plastid-localized Glc6P importer, plays a key role in lipid body formation in Arabidopsis pollen development

Mature pollen of *Arabidopsis*, an oleaginous plant, contains a large number of storage lipid bodies, which are spherical organelles with a size ranging from 0.1 to 2.5 μm and contain a TAG matrix, enclosed by a phospholipid monolayer (PL) with unique embedded proteins including oleosins [51, 52]. The formation of these lipid bodies in pollen is thought to be similar to that in oil seeds [17, 18, 53]. As the first step, potential carbon sources need to be transported into the plastids for the synthesis of acetyl-CoA and then fatty acids (reviewed in [24, 26, 37, 54])

In the non-photosynthetic pollen, transportation of reduced carbons into plastids could be a key step in the control of fatty acid biosynthesis. This study, and previous report [36], demonstrated the importance of GPT1 in fatty acid biosynthesis. GPT1 imports Glc6P into plastids in heterotrophic cells/tissues. Pollen grains of *gpt1* genotype accumulate little or no lipid bodies, suggesting that Glc6P is a major carbon source transported into plastids for the generation of acetyl-CoA and/or reducing equivalent NADPH, essential components of fatty acid biosynthesis. GPT1 is highly expressed in pollen at late developmental stages. In contrast, *GPT2*, the only other GPT in *Arabidopsis*, expresses at a very low level in pollen (Fig 1). In addition, the expression of TPT and PPT in pollen is also relatively low (Fig 1), suggesting that limited amounts of triose phosphate and/or PEP are imported into the non-photosynthetic plastids for fatty acid biosynthesis in pollen. Consistently, mutation of *TPT* gene alone does not result in pollen phenotype and the plants are pretty much normal [55]. It is known that feeding of Glc6P to isolated plastids supports a high rate of fatty acid biosynthesis [56–58], again supporting our conclusion that GPT1 plays an important role in lipid body biogenesis during pollen maturation. It was suggested that the activities and properties of transporters are important in determining the metabolic routes by which carbon is imported into the plastid and utilized for fatty acid synthesis [26]. In the case of Arabidopsis pollen, GPT1 appears to be the key transporter involved.

### *GPT1* expression in pollen is temporally regulated by WRKY2/WRKY34 transcription factors

Mutation of both *WRKY2* and *WRKY34* leads to defective pollen development, reduced pollen viability, and reduced pollen germination, pollen tube growth and transmission [35]. Similar to *gpt1* mutant pollen, *wrky2 wrky34* double mutant pollen also shows a lack of or reduced number of lipid bodies based on Nile blue A staining (Fig 2E and 2F) and BODIPY 505/55 staining (Fig 2G and 2H, S5 Fig), suggesting that the defective pollen development of *wrky2 wrky34* double mutant is related to *GPT1* activation. We analyzed the expression pattern of *GPT1* in pollen development in details, and compared it with the temporal expression of *WRKY2* and *WRKY34*. As shown in the Fig 4B to 4Q of Guan et al paper [35], both WRKY2 and WRKY34 proteins reached their peak levels at BCP stage, preceding the accumulation of GPT1-eYFP. At the MP stage, WRKY34 protein disappears, while WRKY2 protein is still present [35]. This is consistent with the conclusion that *WRKY34* was an early pollen gene enriched in UNMs and BCPs based on expression profiling analysis [59]. Expression profiling revealed that a large number of genes including many transcription factors show spatiotemporal-specific expression [60, 61]. However, the signaling pathway is mostly unclear. In addition, few precedents exist about the direct control of target gene expression by those transcription factors during pollen development.

Based on cellular, molecular, and genetic analyses, we demonstrated that *GPT1* functions downstream of WRKY2/WRKY34 in controlling pollen development. *GPT1* expression in *wrky2 wrky34* double mutant background was compromised (Fig 1 and Fig 5). More importantly, pollen-specific overexpression of *GPT1* could partially rescue the defective pollen phenotypes of *wrky2 wrky34* double mutant (Fig 6). Furthermore, both *GPT1-eYFP* transcript and protein levels were reduced when the W-boxes in the *GPT1* promoter were mutated (Fig 7). Taken together, we conclude that spatiotemporal-specifically expressed WRKY2 and WRKY34 transcription factors target directly the *GPT1* promoter and control its spatiotemporal-specific expression, which specifies the function of undifferentiated proplastids by promoting the storage lipid biosynthesis during pollen maturation.

The partial rescue of *wrky2 wrky34* phenotype by pollen-specific expression of *GPT1* also indicates that these two WRKY transcription factors might be involved in regulating other downstream genes. Besides *GPT1*, WRKY2 and WRKY34 may control the expression of additional genes involved in lipid body biogenesis. As shown in Fig 1, the expression of enzymes in TAG biosynthesis such as *LPAAT2*, *DGAT1*, and *PDAT1* were all reduced. In addition, expression of genes encoding the proteins embedded in the phospholipid monolayer that surrounds oil bodies including *OLE* and *CLO* was also reduced in *wrky2 wrky34* double mutant. At this stage, it is unknown whether all these genes are co-regulated by these two WRKY transcription factors or, alternatively, their expression reduction is a secondary response caused by the lack/reduction of fatty acid biosynthesis. It is interesting to note that DGAT1 and PDAT1 were shown to have overlapping functions in Arabidopsis triacylglycerol biosynthesis and they are essential for normal pollen and seed development [15]. Double *dgat1 pdat1* mutation results in sterile pollen that lacked visible oil bodies, a phenotype similar to that of *gpt1* or *wrky2 wrky34*. The potential regulation of *DGAT1* and *PDAT1* expression by MPK3/MPK6-WRKY2/WRKY34 pathway remains to be examined.

### MPK3/MPK6 cascade-mediated spatiotemporal-specific activation of WRKY transcription factors and *GPT1* expression during pollen development

Expression profiling revealed dynamic changes of gene expression during pollen development [61–63]. Genetic screens have also uncovered a large number of genes encoding transcription factors, receptor-like kinases, and putative peptide ligands involved in various aspects of anther/pollen development (reviewed in [64, 65]). These findings suggest possible signaling pathway(s) from the sensing of extracellular ligands by cell-surface receptors, to the activation of transcription activators/suppressors, to the gene expression reprogramming during pollen development. MAPK cascades are key signaling modules downstream of receptors in plant growth and development [31]. Besides regulation at transcriptional level, WRKY34 is also regulated at the post-translational level, and is phosphorylated by MPK3/MPK6 at the late BCP stage and early TCP stages, and becomes dephosphorylated at the late TCP stage. In addition, genetic analysis demonstrated that the phosphorylation of WRKY34 is important for its biological function in pollen development [35]. It is speculated that WRKY2 is likely subjected to the same post-translational regulation by MPK3/MPK6 based on 1) high homology between WRKY2 and WRKY34, 2) conserved phosphorylation sites, and 3) functional redundancy with WRKY34. However, direct experimental evidence is still lacking. Based on our understanding of the regulation of WRKY33 by MPK3/MPK6 [34] and the high homology of WRKY2/WRKY34 to WRKY33, we speculated that phosphorylation of WRKY2/WRKY34 also changes the transactivation activity of WRKY2/WRKY34 [31]. This is consistent with the fact that the MPK3/MPK6-phosphorylation sites of WRKY2/WRKY34 are within their transactivation domains.

The spatiotemporal phosphorylation of WRKY34 and the accumulation of WRKY2/WRKY34 protein in the vegetative nucleus of BCP stage pollen are consistent with the activation of *GPT1* expression in the vegetative cell at the late stages of pollen development. Furthermore, fluorescent signal from the fully functional GPT1-eYFP fusion became stronger when MPK3 and MPK6 were activated in the gain-of-function system (Fig 8A), and weaker when *MKK4* and *MKK5* were mutated (Fig 8B). In addition, lipid bodies in *mpk3 mpk6* and *mkk4 mkk5* double mutant pollen were significantly reduced (Fig 8C–8F and S13 and S14 Figs). In summary, our data suggest that MKK4/MKK5-MPK3/MPK6 module functions upstream of WRKY2/WRKY34 in regulating the spatiotemporal expression of plastid-localized *GPT1*, an important transporter that translocates Glc6P into pollen plastids for storage lipid biosynthesis during Arabidopsis pollen development. Loss of components in this pathway will reduce the accumulation of storage compounds during pollen maturation process, which negatively impacts pollen viability, pollen germination, and pollen transmission in plant sexual reproduction.

The roles of plastids in heterotrophic cells such as pollen grains are less well understood in comparison to their counterpart, chloroplasts, in photosynthetic cells. Demonstration of an important role of plastidic GPT1 in storage lipid body biogenesis under the control of a MAPK-WRKY signaling pathway highlights the regulation of metabolic activities in plastids by a cytoplasmic/nuclear signaling pathway. The upstream ligand(s) and receptor(s) that activate MPK3/MPK6 are unclear at present, and further research is needed to define the whole signaling pathway. Starch granule and lipid body accumulation during pollen development are critical to pollen functions including pollen germination, pollen tube growth, and successful fertilization. In crop production, reduced yield under environmental stresses is frequently associated with the reduction of storage starch/lipid accumulation in pollen [10, 11]. MPK3/MPK6 cascade is involved in plant response to almost all stresses from both biotic and abiotic sources [32, 33, 46]. As a result, this MAPK cascade may also function as a key integration point where environment factors impinge on the program of pollen development and fitness.

## Materials and methods

### Mutants and transgenic lines

*Arabidopsis thaliana* mutant and transgenic plants related to *MKK4/MKK5*, *MPK3/MPK6*, and *WRKY2/WRKY34* were all in Col-0 ecotype background. Mutant and transgenic lines related to *gpt1*^*+/-*^ were in Ws-2 ecotype. Wild-type plants of Col-0 or Ws-2 ecotype were used as controls depending on the mutants or transgenic plants with which they were compared.

Steroid-inducible promoter-driven tobacco *MEK2*^*DD*^ transgenic Arabidopsis line (*DD*) [43], chemical genetically rescued *mpk3 mpk6* double mutant (*MPK6SR*) [47], and *wrky2 wrky34* double mutant [35] were described previously. Heterozygous *gpt1*^*+/-*^ mutant in Ws-2 background [36] was kindly supplied by Dr. Anja Schneider (Department of Biology I, Ludwig-Maximilian-University). Tilling *mkk4* and *mkk5* single mutants [66] were kindly provided by Dr. Wolfgang Lukowitz (Virginia Tech). Double *mkk4 mkk5* mutant was generated by crossing after removing the *er105* mutant allele.

### Plant growth and treatments

Arabidopsis seeds were surface sterilized. After being imbibed at 4 ^o^C for 3 days, the seeds were plated on half-strength Murashige and Skoog medium with 0.45% Phytagar. Plates were incubated in a tissue culture chamber at 22 ^o^C under continuous light (70 μE m^-2^ s^-1^) for 7 days. Seedlings were then transplanted to soil and grown in a growth chamber with a 14-h-light/10-h-dark cycle.

Dexamethasone (DEX) and 4-amino-1-tert-butyl-3-(1’-naphthyl)pyrazolo [3,4-d]pyrimidine (NA-PP1) were used at final concentrations of 30 μM and 5 μM, respectively. DEX and NA-PP1 stock solutions were prepared in ethanol and DMSO, respectively. Equal volumes of ethanol or DMSO were used as negative controls. For observation of the effect of DEX on the *GPT1-eYFP* expression in the pollen grains of *P*_*GPT1*_:*GPT1-eYFP DD* plants, the inflorescences were sprayed with DEX solution or solvent negative control. After 36 hours, the microspores at the late uninucleate stage were isolated and observed. Application of NA-PP1 inhibitor effectively inhibits the activity of chemical-sensitized MPK6^YG^, giving rise to the activity null double mutant of *MPK3* and *MPK6*. To determine the pollen development and function of pollen grains from *mpk3 mpk6* double mutant plants, we submerged the inflorescences of *MPK6SR* plants in NA-PP1 solution (5 μM) for 10 seconds, and this treatment was repeated every 12 hours. Five days later, the mature pollen was collected, stained with Nile blue A and observed.

### Molecular cloning and transformation

To generate the *GPT1* promoter*-driven GPT1-eYFP* construct (*P*_*GPT1*_:*GPT1-eYFP*), we amplified the *GPT1* genomic DNA by using *GPT1-FP* (5’-AAATGCACATGCTGATGCTATG-3’) and *GPT1-BP* (5’-CTGGTCAGTACGTTTCCAACAA-3’) primer pair. The PCR fragment was cloned into the pBlueScript II KS vector to generate pBS-*P*_*GPT1*_:*GPT1* construct. A Sma I site was added in front of the stop code by PCR amplification of pBS-*GPT1* construct using *GPT1-Sma I-FP* (5’-GGGTGATGCGAAAGACATAAGAGTGTA-3’) and *GPT1-Sma I-BP* (5’-GGGGAGCTTTGCCTGCAAAACAC-3’) primer pair. The DNA was end phosphorylated and ligated to generate pBS-*GPT1-Sma I* construct. The eYFP fragment was then inserted into the Sma I-digested pBS-*P*_*GPT1*_:*GPT1-Sma I* construct to generate pBS-*P*_*GPT1*_:*GPT1-eYFP*. *GPT1* native promoter-driven *GPT1-eYFP* fragment was then cloned into pCambia3300 binary vector using Hind III and Bam HI sites to generate pCambia3300-*P*_*GPT1*_:*GPT1-eYFP*.

To overexpress GPT1-eYFP protein in pollen specifically, we use a strong pollen-specific promoter, *LAT52* [41]. We first introduced a Sma I site before the start code by PCR-amplifying pCambia3300-*P*_*GPT1*_:*GPT1-eYFP* without the *GPT1* promoter using *GPT1-eYFP-Sma I-FP* (5’-GGGATGGTTTTATCGGTGAAGCAAAC-3’) and *GPT1-eYFP-Sma I-BP* (5’-GGGGGATCCACTAGTTCTA-3’) primer pair. *LAT52* promoter fragment was then inserted into the Sma I-digested pCambia3300-*GPT1-eYFP* construct to generate pCambia3300-*LAT52*:*GPT1-eYFP*. To mutate all four W-boxes in the *GPT1* promoter, we divided the pCambia3300*-P*_*GPT1*:_*GPT1-eYFP* construct into four fragments at the sites of W-boxes and amplified each fragment separately using primers with mutated W-boxes sequence. GBclonart Seamless Assembly Kit (Genebank Biosciences Inc. Suzhou, China) was used to assemble the four fragments into the vector to generate pCambia3300*-P*_*GPT1-mW*_:*GPT1-eYFP*.

All the binary vectors were transformed into *Agrobacterium* strain GV3101. Arabidopsis transformation was performed by the floral dip procedure [67], and transformants were identified by screening for BASTA resistance. *P*_*GPT1*_:*GPT1-eYFP*^*+/-*^ *gpt1* plants were obtained by crossing *P*_*GPT1*_:*GPT1-eYFP gpt1* plants with *gpt1*^*+/-*^ heterozygous mutant plants. F2 progenies from *P*_*GPT1*_:*GPT1-eYFP*^*+/-*^ *gpt1* F1 plants with either *P*_*GPT1*_:*GPT1-eYFP*^*+/-*^ *gpt1* or *P*_*GPT1*_:*GPT1-eYFP gpt1* genotype were used in our experiments.

### Cytological and phenotypic analyses

Fluorescence microscope was performed with a Nikon Eclipse 80i microscope equipped with a Nikon Intensilight C-HGFI and fluorescence filter sets. Fluorescence signal was recorded using a TRITC (EX 540/25; DM 565; BA 605/55) filter set for propidium iodide (PI), a FITC (EX 465–495; DM 505; BA 515–555) filter set for eYFP, and a DAPI (EX 340–380; DM 400; BA 435–485) filter set for DAPI. Images were captured utilizing the Nikon Digital Camera DS-Fi1c and imaged with NIS Elements 4.1. Pollen viability was examined by staining pollen grains with 2 μg/ml PI [38]. Lipids in pollen grains was stained with 20 μg/ml Nile blue A [68]. For PI and Nile blue A staining, pollen grains were collected from the floral buds at the +1 stage as previously described [35]. DAPI was used to stain vegetative and generative/sperm nuclei and to determine the pollen development stage [35]. Floral buds at each stage were carefully dissected under stereoscope. Anthers were isolated and transferred to a drop of DAPI solution. A fine needle was used to gently break the anthers, and a cover slip was then used to carefully squeeze the anthers to release the pollen. Pollen germination assays were performed as described previously with slight modification [69, 70]. The basic medium was composed of 1 mM KCl, 10 mM CaCl_2_, 0.8 mM MgSO4, 1.5 mM boric acid, 5 mM MES, 10 μm D-myo-inositol, 18% sucrose, 1.5% (w/v) low-melting agar, and the pH was adjusted to 5.8 with KOH.

### BODIPY 505/515 staining of pollen lipid bodies and quantification

BODIPY 505/515 (4, 4-difluro-1, 3, 5, 7-tetramethyl-4-bora-3a, 4-adiaza-s-indacene; Invitrogen Molecular Probes, USA) was dissolved in anhydrous dimethyl sulfoxide (DMSO) as a stock solution at a concentration of 1.0 mg/mL. For Arabidopsis pollen staining, a final concentration of 1.0 μg/mL was used. Lipid droplets in stained pollen were observed using a Nikon Eclipse 80i microscope or a confocal microscope system (Carl Zeiss upright LSM 710 NLO). To quantify the fluorescence intensities of BODIPY 505/515 stained pollen, we first converted the images to grey scale images. The intensity of each pollen grain was then quantified using ImageJ.

### Quantitative RT-PCR analysis

Anthers with pollen at bicellular pollen (BCP) or tricellular pollen (TCP) stage were detached and submerged in 0.3 M mannitol solution. A fine needle was used to gently break the anthers to release the pollen, and the pollen grains (suspended in the mannitol solution) were transferred into a 1.5 mL tube using a glass capillary tube. Pollen grains from 10 flowers of similar stages collected from 3 plants were pooled together in each of the three repeats. After centrifugation at 450 *×g* for 5 min at 4°C, the pollen pellets were washed twice with 0.3 M mannitol solution. Total RNAs were isolated using TRIzol reagent. After DNase treatment, 0.5 μg of total RNA was reversely transcribed, and quantitative PCR analysis was performed using an Eppendorf real-time PCR machine. Relative levels of each transcript were calculated after being normalized to the *EF1α* control.

### Protein extraction and immunoblot analysis

Protein extraction was performed as previously described with modifications [34]. Anthers at mature pollen (MP) stage but before dehiscence were collected from the same plant. Anthers were ground in liquid nitrogen and extracted in 20 μl 1.5 × SDS loading buffer without bromophenol blue dye. Concentrations of protein samples were determined by bicinchoninic acid (BCA) assay suing BSA as standard. Due to the high similarity with GFP [71], eYFP fusion proteins can be detected with a rabbit anti-GFP polyclonal antibody (Abmart). Immunoblot detection of GPT1-eYFP was performed as previously described [72].

### Quantification of pollen size and fluorescence intensity

Images of mature pollen were taken. Length of pollen grains was measured using ImageJ after the scale tool was set to establish a 100 μm reference on the images. To quantify the fluorescence intensities of pollen grains with GPT1-eYFP transgene, we first converted the fluorescence images to grey scale images, and then the intensity of each pollen grain was quantified using ImageJ.

### Statistical analyses

All experiments were repeated independently at least three times, and representative results are shown. For the purpose of calculating percentages of pollen grains with a particular phenotype, at least 80 pollen grains (indicated in the figure legends) were analyzed in each of the repeats in order to obtain an average of the percentages with standard deviation. One-way ANOVA Tukey’s test was used for statistical analysis. One and two asterisks above the columns indicate differences that are statistically significant (*P* ≤ 0.05) and very significant (*P* ≤ 0.01), respectively.

## Supporting information

S1 Text Supplemental Methods. (PDF)

S1 Table Primer pairs used in RT-qPCR. (PDF)

S1 Fig Normal flower and anther development in *gpt1*^*+/-*^ plants. Flowers at different stages were detached from inflorescence stems and two of the flower pedals were removed to reveal the internal flower organs (**A**). Stamens were detached from flowers at the base of filaments (**B**). Flowers, in which anthesis is about to occur, were designated as Stage 0. An open flower right after anthesis was designated +1. Younger flowers/buds were designated using negative numbers. Bar = 1 mm.
(PDF)

S2 Fig Pollen viability assay using double staining with propidium iodide and fluorescein diacetate. Pollen grains from *gpt1*^*+/-*^ plants were stained with both propidium iodide (PI) and fluorescein diacetate (FDA). (**A**) Dead pollen grains fluoresce red after PI staining. (**B**) Live pollen grains fluoresce green after FDA staining. (**C**) Merged image of PI and FDA fluorescence. Occasionally, there was pollen that could not be stained by either dye, as the one indicated by an arrow. Bar = 100 μm.
(PDF)

S3 Fig Mutant *gpt1* pollen grains have large vacuoles (void spaces) and greatly reduced number of lipid bodies. Transmission electron microscopic (TEM) images of Ws-2 (**A and B**) and *gpt1* (**C**) pollen. B is of a high magnification to show the difference between plastids, which is surrounded by a double membrane, and lipid bodies, which have a homogenous interior. L, lipid body; and P, plastid. Bar = 1 μm.
(PDF)

S4 Fig Localization of GPT1-eYFP on plastids. Binary construct with mCherry targeted to plastids (pt-rk CD3-999) was transformed into *P*_*GPT1*_:*GPT1-eYFP* transgenic background. Homozygous T3 plants were used for co-localization experiments. (**A**) Co-localization of GPT1-eYFP and mCherry plastid marker in epidermal cell. (**B**) Localization of GPT1-eYFP in small organelles in pollen. Pollen outline was visualized by FM4-64 staining. Because mCherry plastid marker is driven by 35S dual enhancer promoter, it is not expressed in pollen grain, which makes co-localization experiment in pollen grains impossible. The presence of GPT1-eYFP in organelles with hollow center region is consistent with its localization on plastid membrane reported previously. Bar = 10 μm.
(PDF)

S5 Fig Loss of function of both *WRKY2* and *WRKY34* compromises lipid body accumulation in pollen. (**A**) BODIPY 505/515 staining of lipid bodies in pollen from *P*_*GPT1*_:*GPT1-eYFP*, *P*_*GPT1*_:*GPT1-eYFP wrky2*, *P*_*GPT1*_:*GPT1-eYFP wrky34*, and *P*_*GPT1*_:*GPT1-eYFP wrky2 wrky34*. (**B**) Quantitation of BODIPY 505/515 fluorescence intensity in pollen grains of different genotypes. Fluorescence intensity was quantified by ImageJ, and normalized to that in *P*_*GPT1*_:*GPT1-eYFP*, which was set as 100%. Two independent *P*_*GPT1*_:*GPT1-eYFP* transgenic lines in wild-type and *wrky* single/double mutant backgrounds were obtained and both gave similar results. Results from one of them are shown. Error bars indicate SD (n ≥ 20). **P ≤ 0.01. Bar = 10 μm.
(PDF)

S6 Fig DAPI staining of nuclei in *gpt1* mutant pollen grains at different developmental stages. Pollen grains from Ws-2 or *gpt1*^*+/-*^ plants at different development stages were stained with DAPI and imaged under a fluorescent microscope. Left panels: bright field images to show pollen morphology, and right panels: DAPI staining of pollen grains from the same anthers to show pollen nuclear stage. At TCP and MP stages, smaller pollen grains (indicated by arrowheads), possibly of *gpt1* genotype, could be identified in those from *gpt1*^*+/-*^ plants. UNM, uninucleate microspore; BCP, bicellular pollen; TCP, tricellular pollen; and MP, mature pollen. Bar = 10 μm.
(PDF)

S7 Fig DAPI staining of nuclei in *wrky2 wrky34* mutant pollen grains at different developmental stages. Pollen grains from Col-0 or *wrky2 wrky34* plants at different development stages were stained using DAPI and imaged under a fluorescent microscope. Left panels: bright field images to show pollen morphology, and right panels: DAPI staining of pollen grains from the same anthers to show pollen nuclear stage. UNM, uninucleate microspore; BCP, bicellular pollen; TCP, tricellular pollen; and MP, mature pollen. Bar = 10 μm.
(PDF)

S8 Fig Death of *gpt1* and *wrky2 wrky34* pollen occurs at the late development and maturation stages. Pollen grains from *P*_*GPT1*_:*GPT1-eYFP*^*+/-*^ *gpt1* (**A**) and *wrky2 wrky34* (**B**) plants at different pollen development stages were stained with PI and imaged under a fluorescent microscope. Dead pollen grains with red fluorescence and live pollen grains were counted. In panel A, pollen grains of different genotypes, fluorescent-rescued *P*_*GPT1*_:*GPT1-eYFP gpt1* pollen (equivalent to wild-type) and non-fluorescent *gpt1* mutant pollen grains, from *P*_*GPT1*_:*GPT1-eYFP*^*+/-*^ *gpt1* plants were quantified separately. At least 100 pollen grains were counted in each repeat. Error bars indicate SD (n = 3). **P ≤ 0.01.
(PDF)

S9 Fig Starch accumulation in pollen grains from Ws-2 and *gpt1*^*+/-*^ plants at different developmental stages. Pollen grains from Ws-2 and *gpt1*^*+/-*^ plants were stained with Lugol's iodine solution and imaged. Top panels: Lugol's iodine staining to show starch accumulation; and bottom panels: DAPI staining of pollen grains from the same anthers to determine pollen nuclear stage. BCP, bicellular pollen; TCP, tricellular pollen; and MP, mature pollen. Bar = 10 μm.
(PDF)

S10 Fig Starch accumulation during pollen development in Col-0 and *wrky2 wrky34* double mutant plants. Pollen grains from Col-0 and *wrky2 wrky34* plants were stained with Lugol's iodine solution and imaged. Top panels: Lugol's iodine staining to show starch accumulation; and bottom panels: DAPI staining of pollen grains from the same anthers to determine pollen nuclear stage. BCP, bicellular pollen; TCP, tricellular pollen; and MP, mature pollen. Bar = 10 μm.
(PDF)

S11 Fig Pollen-specific overexpression of *GPT1-eYFP* enhances the accumulation of lipid bodies in *wrky2 wrky34* pollen. (**A**) BODIPY 505/515 staining of lipid bodies in pollen grains from *wrky2 wrky34* and *P*_*LAT52*_:*GPT1-eYFP wrky2 wrky34* plants. (**B**) Quantitation of BODIPY 505/515 fluorescence intensity in pollen grains from *wrky2 wrky34* and *P*_*LAT52*_:*GPT1-eYFP wrky2 wrky34* plants. Fluorescence intensity was quantified using ImageJ and normalized to that in *wrky2 wrky34*, which was set as 100%. Three independent *P*_*LAT52*_:*GPT1-eYFP* transgenic lines in *wrky2 wrky34* background were analyzed and all gave similar results. Results from one of the three lines are shown. Error bars indicate SD (n ≥ 35). **P ≤ 0.01. Bar = 10 μm.
(PDF)

S12 Fig WRKY34 binds to *GPT1* promoter in yeast one-hybrid assay. (**A**) Four W-boxes in the *GPT1* promoter were combined to a 106-nucleotide-long *GPT1* promoter fragment (*P*_*GPT1*_), which was used for DNA-binding assay in yeast. W-boxes are marked in black. Red-colored letters indicate mutated nucleotides in the W boxes of *GPT1* promoter fragment (*mP*_*GPT1*_). (**B**) Yeast was co-transformed with a reporter vector containing the promoter fragment of *P*_*GPT1*_ or *mP*_*GPT1*_ fused to a *HIS2* reporter gene, and an effector vector containing WRKY34 fused to a GAL4 activation domain. Transformants were selected on double dropout medium (SD-Leu-Trp) and then plated on triple dropout medium (SD-Leu-Trp-His) to test binding. 3-amino-1, 2, 4-triazole (3-AT, 90 mM) was included to suppress background growth.
(PDF)

S13 Fig Loss of function of both *MKK4* and *MKK5* compromises lipid body accumulation in mature pollen. (**A**) BODIPY 505/515 staining of lipid bodies in pollen grains from Col-0, *mkk4*, *mkk5*, and *mkk4 mkk5* plants. (**B**) Quantitation of BODIPY 505/515 staining of pollen grains from Col-0, *mkk4*, *mkk5*, and *mkk4 mkk5* plants. The intensity of fluorescence was quantified using ImageJ, and normalized to that in Col-0 control, which was set as 100%. Error bars indicate SD (n ≥ 20). **P ≤ 0.01. Bar = 10 μm.
(PDF)

S14 Fig Loss of function of *MPK3* and *MPK6* compromises lipid body accumulation in mature pollen. (**A**) BODIPY 505/515 staining of lipid bodies in pollen grains from chemical genetically rescued *MPK6SR* plants treated with either DMSO solvent or NA-PP1. (**B**) Quantitation of BODIPY 505/515 fluorescence intensity in pollen grains from DMSO- or NA-PP1-treated *MPK6SR* plants. The intensity of BODIPY fluorescence was quantified using ImageJ and normalized to that in the DMSO-treated control, which was set as 100%. Error bars indicate SD (n ≥ 25). **P ≤ 0.01. Bar = 10 μm.
(PDF)
