# Supplementary material for: Regulation of pollen lipid body biogenesis by MAP kinases and downstream WRKY transcription factors in Arabidopsis
Source: PLoS Genet. 2018 Dec 26;14(12):e1007880. doi: 10.1371/journal.pgen.1007880 (PMC6324818; doi:10.1371/journal.pgen.1007880)
Supplement: S1 Text — (PDF) [file pgen.1007880.s001.pdf]

Supplemental Methods

Starch staining

For starch staining of pollen grains, inflorescences were first fixed in FAA solution (50% ethanol: acetic acid: formaldehyde, 18:1:1, v/v/v). Flower buds with pollen grains at different developmental stages were then isolated. Pollen grains from the same anther were stained with either Lugol's iodine solution (1% I_2-KI) for starch detection or DAPI for determining the pollen development stage. Lugol's iodine staining was performed as previously described (Su et al., 2017).

Yeast one-hybrid assay

Wild-type *GPT1* promoter fragment containing all four W boxes was introduced in front of *HIS2* reporter gene using inverted PCR amplification of *pHIS2.1* vector with *P_{GPT1}-pHIS2-FP* (5'-CAAAACTGACTCAATTCATAATTTTAGATTCCAGGTCAAACT GTGATCCAggggagctcacgc-3') and *P_{GPT1}-pHIS2-BP* (5'-ATTGATATCAATTGACTAT GAAATCATTTGGATCACAGTTTGACCTGGAATCTAAAgggaattcgccct-3') primer pair. Lower case letters indicate sequence that matches the pHIS2.1 vector. *GPT1* promoter with all four W-boxes mutated was generated similarly using *mP_{GPT1}-pHIS2-FP* (5'-CAAAACTGAATCAATTCATAATTTTAGATTCCAGTTCAAACTGTGATCCAgggg agctcacgc-3') and *mP_{GPT1}-pHIS2-BP* (5'-ATTGATATCAATTGAATATGAAATCATTT GGATCACAGTTTGAACTGGAATCTAAAgggaattcgccct-3') primer pair. PCR products were then end-phosphorylated and ligated before being transformation into *E. coli*. All constructs were confirmed by sequencing. WRKY34 coding region was PCR-amplified using *WRKY34 CDS-FP* (5'-CCGGAATTCATGGCTGGTATTGATAATAAAGCTGC-3') and *WRKY34 CDS-BP* (5'-TCCCCCGGGTCATATCTGTCGTAATCTACTCAACATC-3'), and cloned into *pGAD7-rec2* vector (CLONTECH), creating a translational fusion of GAL4 activation domain and WRKY34 transcription factor. Yeast one-hybrid assays were performed as described before (Yan et al., 2016). Briefly, yeast competent cells (Y187 strain) were prepared and transformed according to the Clontech Yeast Protocols Handbook. Transformations were plated on SD media-Leu-Trp and incubated at 28 ^{o}C for 4 days to select co-transformants. Transformed yeast cells were subsequently grown in SD-Trp-Leu liquid media overnight and adjusted to OD_{600} of 0.1. The suspensions were then spotted on SD-Trp-Leu and SD-Trp-Leu-His media plates supplemented with 90 mM 3-amino-1, 2, 4-triazole (3-AT) (Sigma-Aldrich). The plates were incubated for 6 days at 28 ^{o}C.

Co-localization experiment

For co-localization study, we transformed plastid marker construct, *pt-rk CD3-999* (Nelson et al., 2007), into *P_{GPT1}:GPT1-eYFP* transgenic background. Homozygous T3 plants were used for co-localization experiments. Because this plastid marker is driven

by 35S dual enhancer promoter, it is not expressed in pollen grains, which makes co-localization experiment in pollen grains impossible. As a result, we used epidermal cells to study the co-localization of GPT1-eYFP and mCherry plastid marker.

Supplemental References

Nelson, B.K., Cai, X., and Nebenfuhr, A. (2007). A multicolored set of in vivo organelle markers for co-localization studies in Arabidopsis and other plants. Plant J. **51:** 1126-1136.

Su, J., Zhang, M., Zhang, L., Sun, T., Liu, Y., Lukowitz, W., Xu, J., and Zhang, S. (2017). Pathogen-responsive MPK3/MPK6 cascade and abscisic acid function interdependently to regulate stomatal immunity in *Arabidopsis.* Plant Cell **29:** 526-542.

Yan, J.Y., Li, C.X., Sun, L., Ren, J.Y., Li, G.X., Ding, Z.J., and Zheng, S.J. (2016). A WRKY transcription factor regulates Fe translocation under Fe deficiency. Plant Physiol. **171:** 2017-2027.
