# Supplementary material for: Regulation of pollen lipid body biogenesis by MAP kinases and downstream WRKY transcription factors in Arabidopsis
Source: PLoS Genet. 2018 Dec 26;14(12):e1007880. doi: 10.1371/journal.pgen.1007880 (PMC6324818; doi:10.1371/journal.pgen.1007880)
Supplement: S1 Table — (PDF) [file pgen.1007880.s002.pdf]

Supplemental Table S1: Primer pairs used in RT-qPCR.

Gene	Forward (5'-3')	Backward (5'-3')
GPT1	GAGCTCCTGACTTCGGTTAAAA	ATCTTCTCATTCCCCCAAATTC
GPT2	GGTGAGATCAACATCGGAAAAT	TTAGCCTGTCTTGTGCAGAATC
TPT	GATCGGTTTCTCCATCGTTATC	CTCTTCTTCGATCTTGGCCTTA
PPT1	ACATTCTGTCGGAAACTGTGTG	GTATAAGAAGACACCCGCAAGG
G6PDH1	AGATGCAGCATGGGATCTATTC	CAAGATCTCCCCATCTCACATT
G6PDH3	GGTGAACGCAGATTGTTCATAA	ACGACTGCCGTAAGGATAGAAT
6PGPDH1	CCTGGAATGTGTGCTAGTCTTG	AGGACGATCGGTTCTCTCATAA
TKL1	CCATCGGATGTATCAGCTAGAGT	TTGTAGAGTAAGGGTGCTGGTG
PDH-E1α	ATAGACGAGTTGGTGGAGGAAG	TAAACTTGGGGTCCTCACATCT
PDH-E1β2	TTTGATCGTGGAGGAGTGTATG	GCGTAAGGTGTAGGAACGTCTT
α-CT	CAGATCAAGCAGAAGATTGCAG	TCGTCATCATCTTCCTTCACAC
BCCP2	TGAACCCCCTTTTGTAAAGGTT	AGCCAGTAGTTCCATGATGGTT
KAS III	ATTTCCCACCAGAGAGAGTCAT	GGATGTCGCTATGGTATGTCCT
KAR1	AAGGTGCGAGCAGGAATATAAA	TACCGTCCTAATGGGATTGTTC
HAD2	CTGGTGATGAGGATGACACTTG	TTTCCCATAGCCATCAAGAACT
ENR1	AAGCCATTGGGTTCATAGACAC	ACATATATGGTTGCACCGGTTA
KAS II	CCAGACAGTGGAGTGGATACAA	GAGCAAAAATGATGCTGGAGTT
SAD4	GTGGAATGTGGAGACTTTGACA	AATGGTATGTTTTTGGCAGCTT
FATA1	GAATGTCAACAAGACGATGTGG	ATCTCCTGACCATCTCCAGACA
GPAT6	ACGTTCCTCAAACAGATTCCAG	GAAATTGGTGCACTCAAAACCT
GPAT9	ATTTGCAGAGAGGGTCAGAGAC	ACTCTGTTGCTTGCGTTCACTA
LPAAT2	TTCTTCATGGAAAGGTATCACG	GATTCTGGGTGGTGATTGTCTT
DGAT1	GTTCCTTGTCGTCTCTTCAAGC	GTTGTCCGAAAATGCAGAAGAT
PDAT1	CCATGTTGATATCATGGGAAAC	ATACGCTCCGACCATTCAAATA
OLE5	CTTAACGGTCTCCGGATTTCTT	CGTAATCTTTGACGTGAGATGC
CLO4	AACGAGGAACTCAAACAACTCC	GCAGCTCTCACTGTGTCTTTGT
