# Supplementary material for: Regulation of pollen lipid body biogenesis by MAP kinases and downstream WRKY transcription factors in Arabidopsis
Source: PLoS Genet. 2018 Dec 26;14(12):e1007880. doi: 10.1371/journal.pgen.1007880 (PMC6324818; doi:10.1371/journal.pgen.1007880)
Supplement: S1 Fig — Flowers at different stages were detached from inflorescence stems and two of the flower pedals were removed to reveal the internal flower organs (A). Stamens were detached from flowers at the base of filaments (B). Flowers, in which anthesis is about to occur, were designated as Stage 0. An open flower right after anthesis was designated +1. Younger flowers/buds were designated using negative numbers. Bar = 1 mm. (PDF) [file pgen.1007880.s003.pdf]

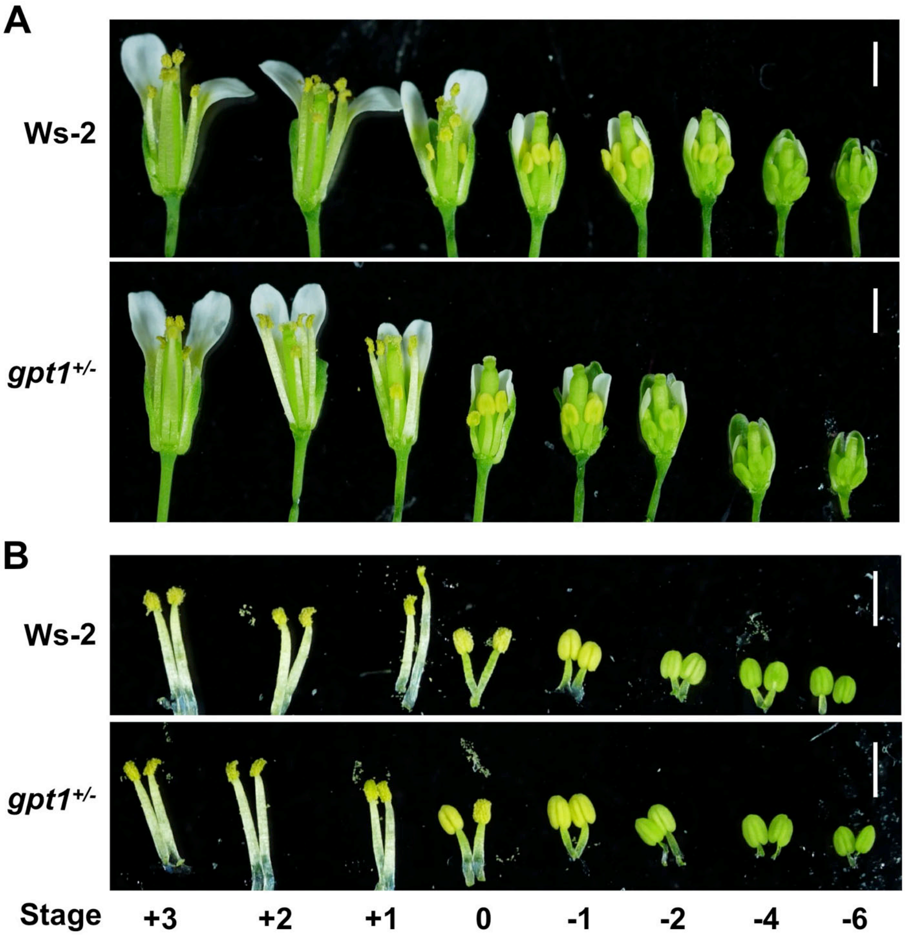

Supplemental Figure S1. Normal flower and anther development in *gpt1*$^{+/-}$ plants.
Flowers at different stages were detached from inflorescence stems and two of the flower pedals were removed to reveal the internal flower organs (**A**). Stamens were detached from flowers at the base of filaments (**B**). Flowers, in which anthesis is about to occur, were designated as Stage 0. An open flower right after anthesis was designated +1. Younger flowers/buds were designated using negative numbers. Bar = 1 mm.
