# Supplementary material for: Regulation of pollen lipid body biogenesis by MAP kinases and downstream WRKY transcription factors in Arabidopsis
Source: PLoS Genet. 2018 Dec 26;14(12):e1007880. doi: 10.1371/journal.pgen.1007880 (PMC6324818; doi:10.1371/journal.pgen.1007880)
Supplement: S2 Fig — Pollen grains from gpt1+/- plants were stained with both propidium iodide (PI) and fluorescein diacetate (FDA). (A) Dead pollen grains fluoresce red after PI staining. (B) Live pollen grains fluoresce green after FDA staining. (C) Merged image of PI and FDA fluorescence. Occasionally, there was pollen that could not be stained by either dye, as the one indicated by an arrow. Bar = 100 μm. (PDF) [file pgen.1007880.s004.pdf]

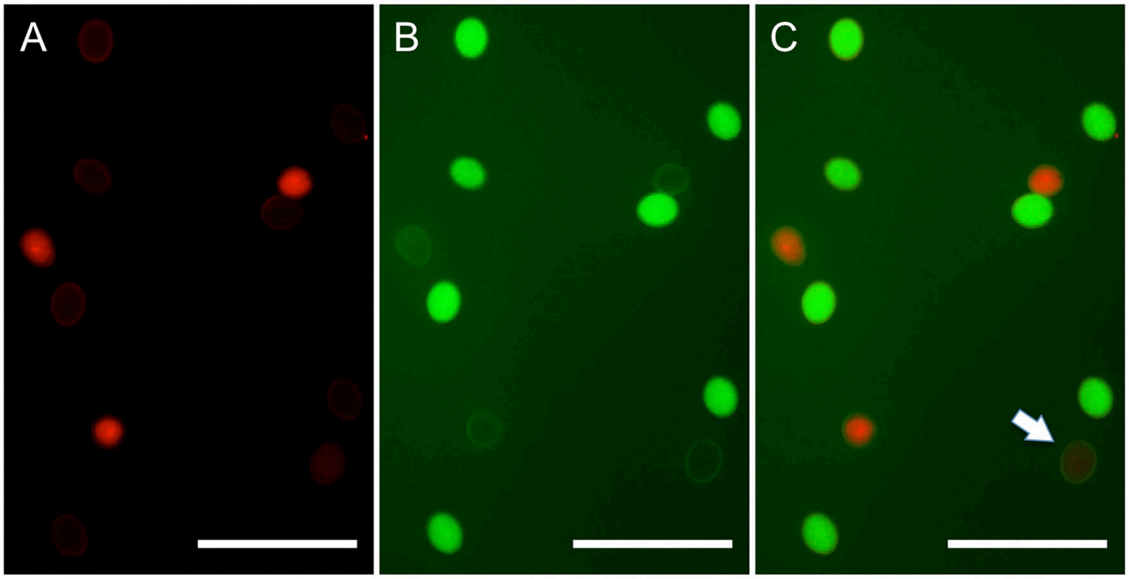

Supplemental Figure S2. Pollen viability assay using double staining with propidium iodide and fluorescein diacetate.

Pollen grains from *gpt1*$^{+/-}$ plants were stained with both propidium iodide (PI) and fluorescein diacetate (FDA). (**A**) Dead pollen grains fluoresce red after PI staining. (**B**) Live pollen grains fluoresce green after FDA staining. (**C**) Merged image of PI and FDA fluorescence. Occasionally, there was pollen that could not be stained by either dye, as the one indicated by an arrow. Bar = 100 μm.
