# Supplementary material for: Regulation of pollen lipid body biogenesis by MAP kinases and downstream WRKY transcription factors in Arabidopsis
Source: PLoS Genet. 2018 Dec 26;14(12):e1007880. doi: 10.1371/journal.pgen.1007880 (PMC6324818; doi:10.1371/journal.pgen.1007880)
Supplement: S3 Fig — Transmission electron microscopic (TEM) images of Ws-2 (A and B) and gpt1 (C) pollen. B is of a high magnification to show the difference between plastids, which is surrounded by a double membrane, and lipid bodies, which have a homogenous interior. L, lipid body; and P, plastid. Bar = 1 μm. (PDF) [file pgen.1007880.s005.pdf]

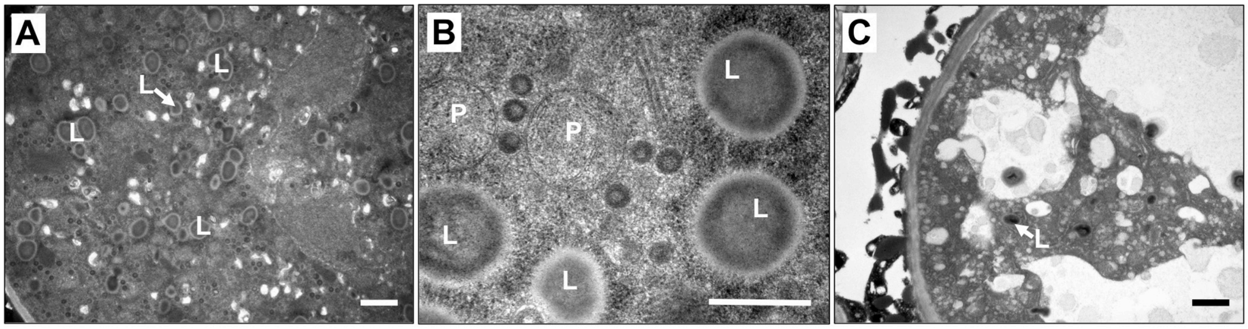

Supplemental Figure S3. Mutant *gpt1* pollen grains have large vacuoles (void spaces) and greatly reduced number of lipid bodies.

Transmission electron microscopic (TEM) images of Ws-2 (**A and B**) and *gpt1* (**C**) pollen. B is of a high magnification to show the difference between plastids, which is surrounded by a double membrane, and lipid bodies, which have a homogenous interior. L, lipid body; and P, plastid. Bar = 1 μm.
