# Supplementary material for: Regulation of pollen lipid body biogenesis by MAP kinases and downstream WRKY transcription factors in Arabidopsis
Source: PLoS Genet. 2018 Dec 26;14(12):e1007880. doi: 10.1371/journal.pgen.1007880 (PMC6324818; doi:10.1371/journal.pgen.1007880)
Supplement: S4 Fig — Binary construct with mCherry targeted to plastids (pt-rk CD3-999) was transformed into PGPT1:GPT1-eYFP transgenic background. Homozygous T3 plants were used for co-localization experiments. (A) Co-localization of GPT1-eYFP and mCherry plastid marker in epidermal cell. (B) Localization of GPT1-eYFP in small organelles in pollen. Pollen outline was visualized by FM4-64 staining. Because mCherry plastid marker is driven by 35S dual enhancer promoter, it is not expressed in pollen grain, which makes co-localization experiment in pollen grains impossible. The presence of GPT1-eYFP in organelles with hollow center region is consistent with its localization on plastid membrane reported previously. Bar = 10 μm. (PDF) [file pgen.1007880.s006.pdf]

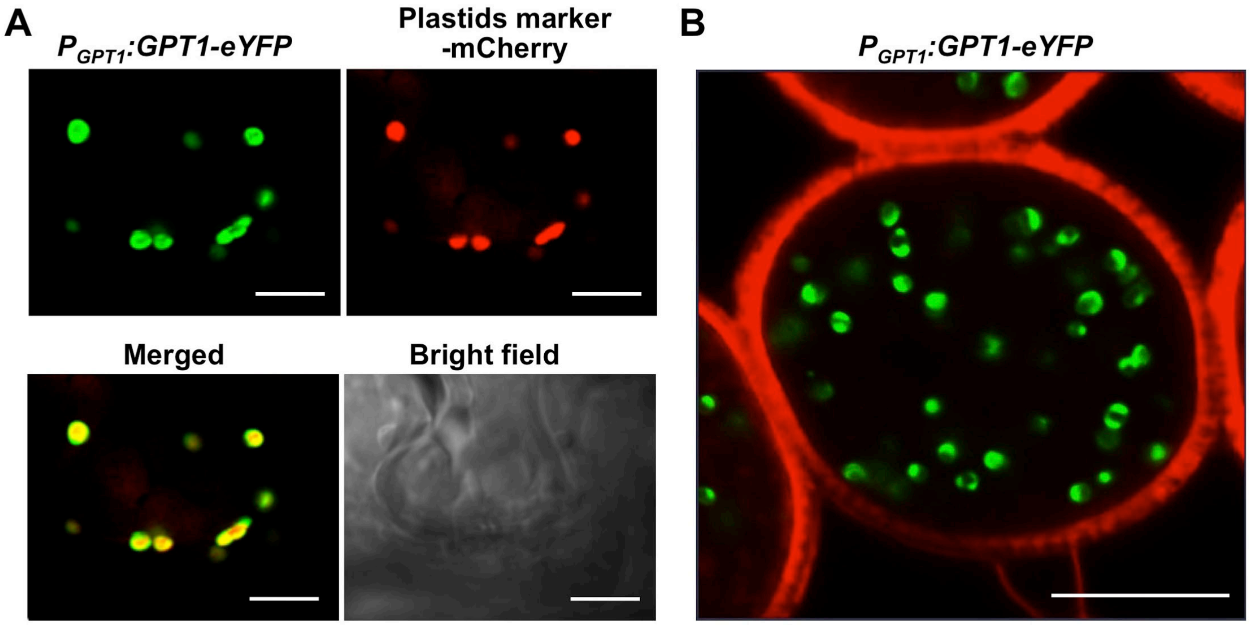

Supplemental Figure S4. Localization of GPT1-eYFP on plastids.
Binary construct with mCherry targeted to plastids (pt-rk CD3-999) was transformed into *P_{GPT1}:GPT1-eYFP* transgenic background. Homozygous T3 plants were used for co-localization experiments. (**A**) Co-localization of GPT1-eYFP and mCherry plastid marker in epidermal cell. (**B**) Localization of GPT1-eYFP in small organelles in pollen. Pollen outline was visualized by FM4-64 staining. Because mCherry plastid marker is driven by 35S dual enhancer promoter, it is not expressed in pollen grain, which makes co-localization experiment in pollen grains impossible. The presence of GPT1-eYFP in organelles with hollow center region is consistent with its localization on plastid membrane reported previously. Bar = 10 μm.
