# Supplementary material for: Regulation of pollen lipid body biogenesis by MAP kinases and downstream WRKY transcription factors in Arabidopsis
Source: PLoS Genet. 2018 Dec 26;14(12):e1007880. doi: 10.1371/journal.pgen.1007880 (PMC6324818; doi:10.1371/journal.pgen.1007880)
Supplement: S5 Fig — (A) BODIPY 505/515 staining of lipid bodies in pollen from PGPT1:GPT1-eYFP, PGPT1:GPT1-eYFP wrky2, PGPT1:GPT1-eYFP wrky34, and PGPT1:GPT1-eYFP wrky2 wrky34. (B) Quantitation of BODIPY 505/515 fluorescence intensity in pollen grains of different genotypes. Fluorescence intensity was quantified by ImageJ, and normalized to that in PGPT1:GPT1-eYFP, which was set as 100%. Two independent PGPT1:GPT1-eYFP transgenic lines in wild-type and wrky single/double mutant backgrounds were obtained and both gave similar results. Results from one of them are shown. Error bars indicate SD (n ≥ 20). **P ≤ 0.01. Bar = 10 μm. (PDF) [file pgen.1007880.s007.pdf]

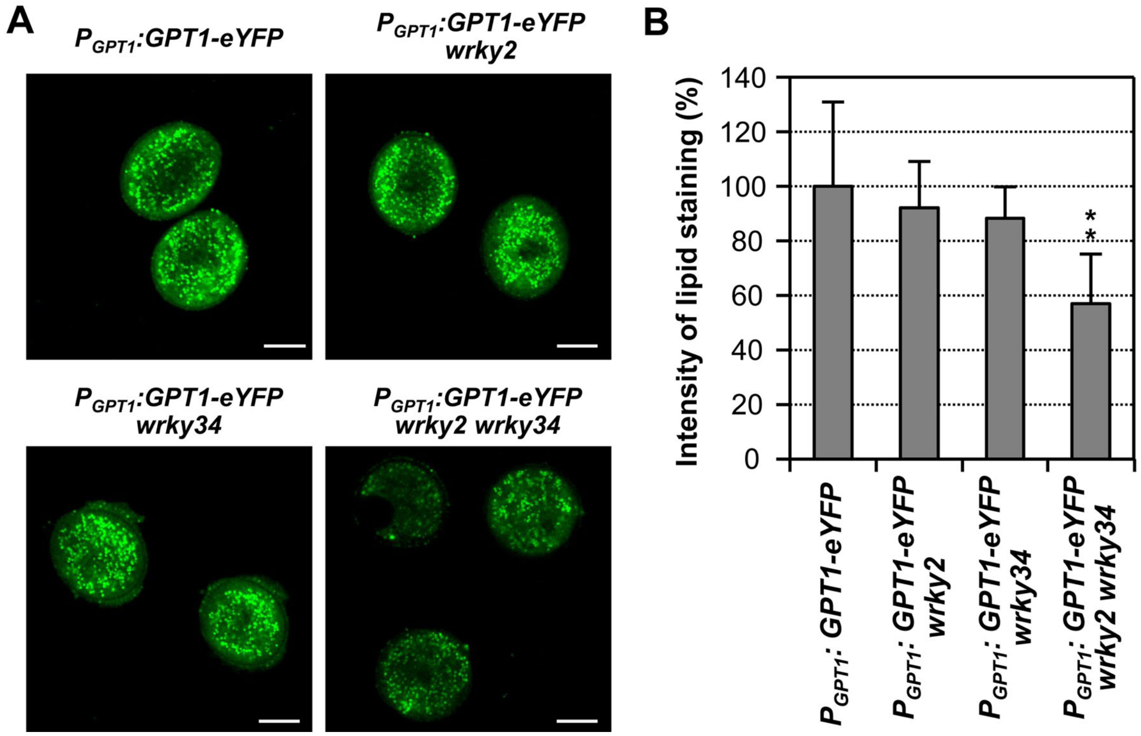

Supplemental Figure S5. Loss of function of both *WRKY2* and *WRKY34* compromises lipid body accumulation in pollen.

(**A**) BODIPY 505/515 staining of lipid bodies in pollen from *P_{GPT1}:GPT1-eYFP*, *P_{GPT1}:GPT1-eYFP wrky2*, *P_{GPT1}:GPT1-eYFP wrky34*, and *P_{GPT1}:GPT1-eYFP wrky2 wrky34*. (**B**) Quantitation of BODIPY 505/515 fluorescence intensity in pollen grains of different genotypes. Fluorescence intensity was quantified by ImageJ, and normalized to that in *P_{GPT1}:GPT1-eYFP*, which was set as 100%. Two independent *P_{GPT1}:GPT1-eYFP* transgenic lines in wild-type and *wrky* single/double mutant backgrounds were obtained and both gave similar results. Results from one of them are shown. Error bars indicate SD (n ≥ 20). **$P \leq 0.01$. Bar = 10 μm.
