# Supplementary material for: Regulation of pollen lipid body biogenesis by MAP kinases and downstream WRKY transcription factors in Arabidopsis
Source: PLoS Genet. 2018 Dec 26;14(12):e1007880. doi: 10.1371/journal.pgen.1007880 (PMC6324818; doi:10.1371/journal.pgen.1007880)
Supplement: S6 Fig — Pollen grains from Ws-2 or gpt1+/- plants at different development stages were stained with DAPI and imaged under a fluorescent microscope. Left panels: bright field images to show pollen morphology, and right panels: DAPI staining of pollen grains from the same anthers to show pollen nuclear stage. At TCP and MP stages, smaller pollen grains (indicated by arrowheads), possibly of gpt1 genotype, could be identified in those from gpt1+/- plants. UNM, uninucleate microspore; BCP, bicellular pollen; TCP, tricellular pollen; and MP, mature pollen. Bar = 10 μm. (PDF) [file pgen.1007880.s008.pdf]

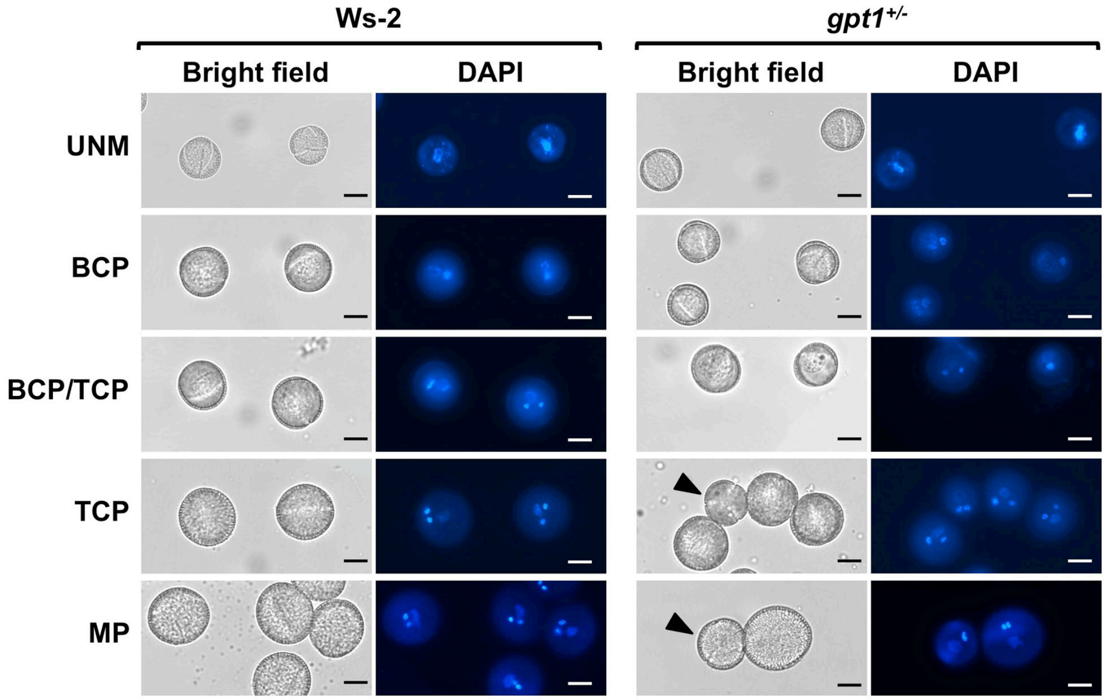

Supplemental Figure S6. DAPI staining of nuclei in *gpt1* mutant pollen grains at different developmental stages.

Pollen grains from Ws-2 or *gpt1*$^{+/-}$ plants at different development stages were stained with DAPI and imaged under a fluorescent microscope. Left panels: bright field images to show pollen morphology, and right panels: DAPI staining of pollen grains from the same anthers to show pollen nuclear stage. At TCP and MP stages, smaller pollen grains (indicated by arrowheads), possibly of *gpt1* genotype, could be identified in those from *gpt1*$^{+/-}$ plants. UNM, uninucleate microspore; BCP, bicellular pollen; TCP, tricellular pollen; and MP, mature pollen. Bar = 10 μm.
