# Supplementary material for: Regulation of pollen lipid body biogenesis by MAP kinases and downstream WRKY transcription factors in Arabidopsis
Source: PLoS Genet. 2018 Dec 26;14(12):e1007880. doi: 10.1371/journal.pgen.1007880 (PMC6324818; doi:10.1371/journal.pgen.1007880)
Supplement: S7 Fig — Pollen grains from Col-0 or wrky2 wrky34 plants at different development stages were stained using DAPI and imaged under a fluorescent microscope. Left panels: bright field images to show pollen morphology, and right panels: DAPI staining of pollen grains from the same anthers to show pollen nuclear stage. UNM, uninucleate microspore; BCP, bicellular pollen; TCP, tricellular pollen; and MP, mature pollen. Bar = 10 μm. (PDF) [file pgen.1007880.s009.pdf]

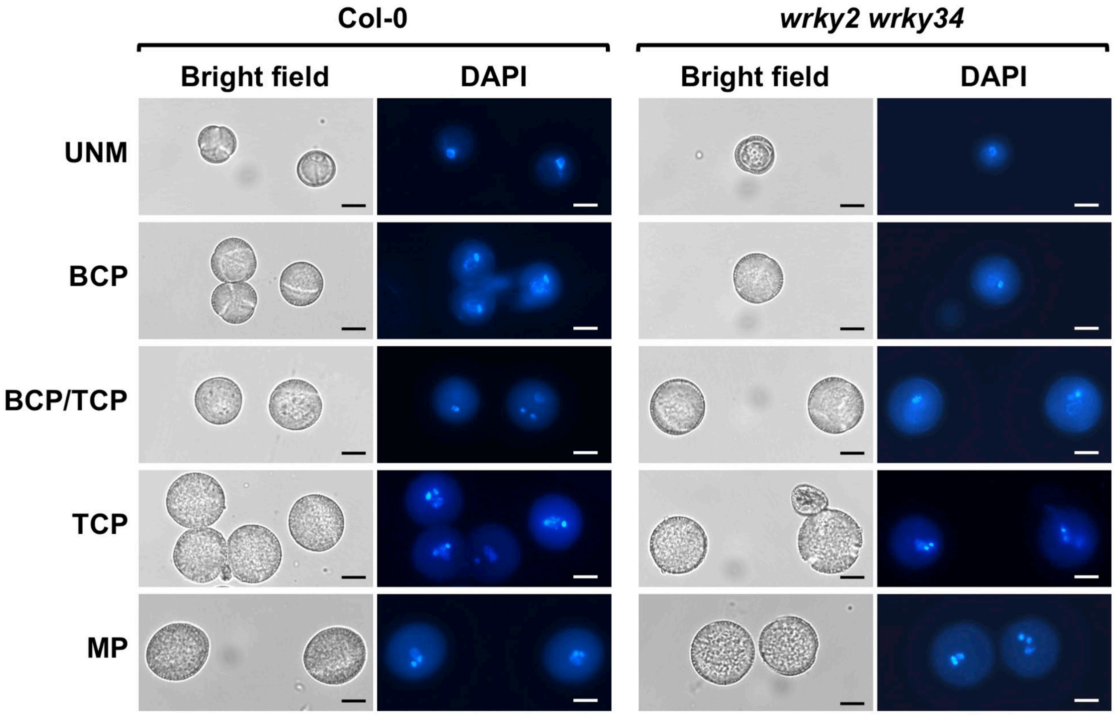

Supplemental Figure S7. DAPI staining of nuclei in *wrky2 wrky34* mutant pollen grains at different developmental stages.

Pollen grains from Col-0 or *wrky2 wrky34* plants at different development stages were stained using DAPI and imaged under a fluorescent microscope. Left panels: bright field images to show pollen morphology, and right panels: DAPI staining of pollen grains from the same anthers to show pollen nuclear stage. UNM, uninucleate microspore; BCP, bicellular pollen; TCP, tricellular pollen; and MP, mature pollen. Bar = 10 µm.
