# Supplementary material for: Regulation of pollen lipid body biogenesis by MAP kinases and downstream WRKY transcription factors in Arabidopsis
Source: PLoS Genet. 2018 Dec 26;14(12):e1007880. doi: 10.1371/journal.pgen.1007880 (PMC6324818; doi:10.1371/journal.pgen.1007880)
Supplement: S8 Fig — Pollen grains from PGPT1:GPT1-eYFP+/- gpt1 (A) and wrky2 wrky34 (B) plants at different pollen development stages were stained with PI and imaged under a fluorescent microscope. Dead pollen grains with red fluorescence and live pollen grains were counted. In panel A, pollen grains of different genotypes, fluorescent-rescued PGPT1:GPT1-eYFP gpt1 pollen (equivalent to wild-type) and non-fluorescent gpt1 mutant pollen grains, from PGPT1:GPT1-eYFP+/- gpt1 plants were quantified separately. At least 100 pollen grains were counted in each repeat. Error bars indicate SD (n = 3). **P ≤ 0.01. (PDF) [file pgen.1007880.s010.pdf]

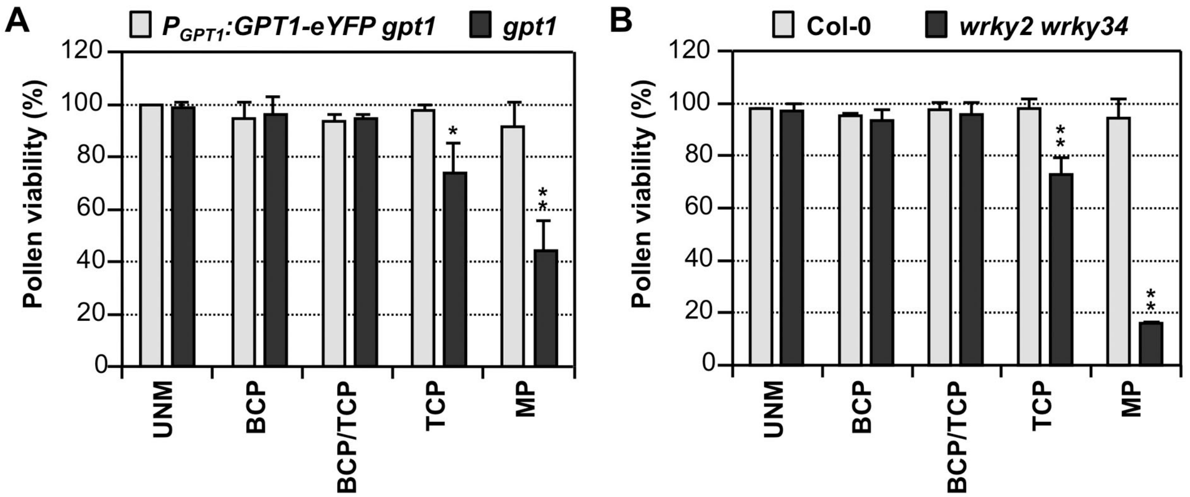


**Supplemental Figure S8.** Death of *gpt1* and *wrky2 wrky34* pollen occurs at the late development and maturation stages.

Pollen grains from *$P_{GPT1}$:GPT1-eYFP$^{+/-}$ gpt1* (**A**) and *wrky2 wrky34* (**B**) plants at different pollen development stages were stained with PI and imaged under a fluorescent microscope. Dead pollen grains with red fluorescence and live pollen grains were counted. In panel A, pollen grains of different genotypes, fluorescent-rescued *$P_{GPT1}$:GPT1-eYFP gpt1* pollen (equivalent to wild-type) and non-fluorescent *gpt1* mutant pollen grains, from *$P_{GPT1}$:GPT1-eYFP$^{+/-}$ gpt1* plants were quantified separately. At least 100 pollen grains were counted in each repeat. Error bars indicate SD (n = 3). $^{**}P \leq 0.01$.
