# Supplementary material for: Regulation of pollen lipid body biogenesis by MAP kinases and downstream WRKY transcription factors in Arabidopsis
Source: PLoS Genet. 2018 Dec 26;14(12):e1007880. doi: 10.1371/journal.pgen.1007880 (PMC6324818; doi:10.1371/journal.pgen.1007880)
Supplement: S9 Fig — Pollen grains from Ws-2 and gpt1+/- plants were stained with Lugol's iodine solution and imaged. Top panels: Lugol's iodine staining to show starch accumulation; and bottom panels: DAPI staining of pollen grains from the same anthers to determine pollen nuclear stage. BCP, bicellular pollen; TCP, tricellular pollen; and MP, mature pollen. Bar = 10 μm. (PDF) [file pgen.1007880.s011.pdf]

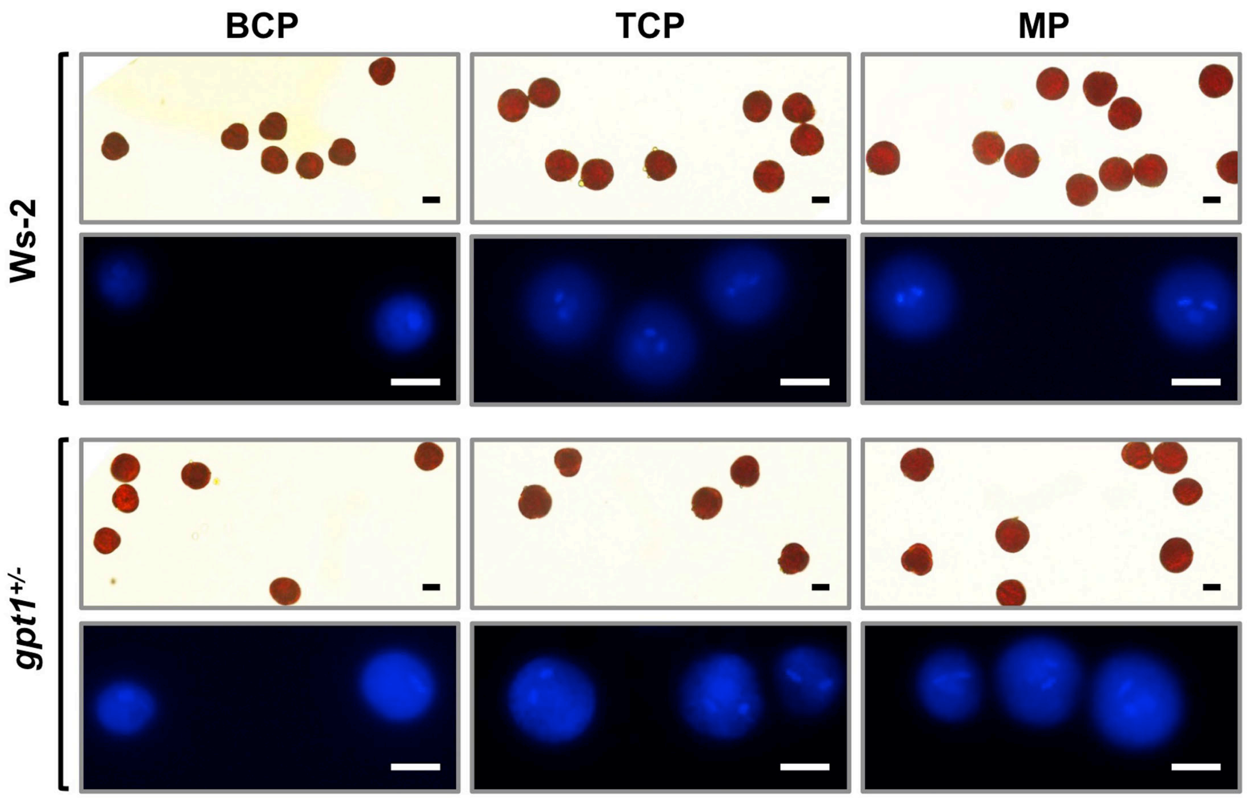

Supplemental Figure S9. Starch accumulation in pollen grains from Ws-2 and *gpt1*$^{+/-}$ plants at different developmental stages.

Pollen grains from Ws-2 and *gpt1*$^{+/-}$ plants were stained with Lugol's iodine solution and imaged. Top panels: Lugol's iodine staining to show starch accumulation; and bottom panels: DAPI staining of pollen grains from the same anthers to determine pollen nuclear stage. BCP, bicellular pollen; TCP, tricellular pollen; and MP, mature pollen. Bar = 10 μm.
