# Supplementary material for: Regulation of pollen lipid body biogenesis by MAP kinases and downstream WRKY transcription factors in Arabidopsis
Source: PLoS Genet. 2018 Dec 26;14(12):e1007880. doi: 10.1371/journal.pgen.1007880 (PMC6324818; doi:10.1371/journal.pgen.1007880)
Supplement: S10 Fig — Pollen grains from Col-0 and wrky2 wrky34 plants were stained with Lugol's iodine solution and imaged. Top panels: Lugol's iodine staining to show starch accumulation; and bottom panels: DAPI staining of pollen grains from the same anthers to determine pollen nuclear stage. BCP, bicellular pollen; TCP, tricellular pollen; and MP, mature pollen. Bar = 10 μm. (PDF) [file pgen.1007880.s012.pdf]

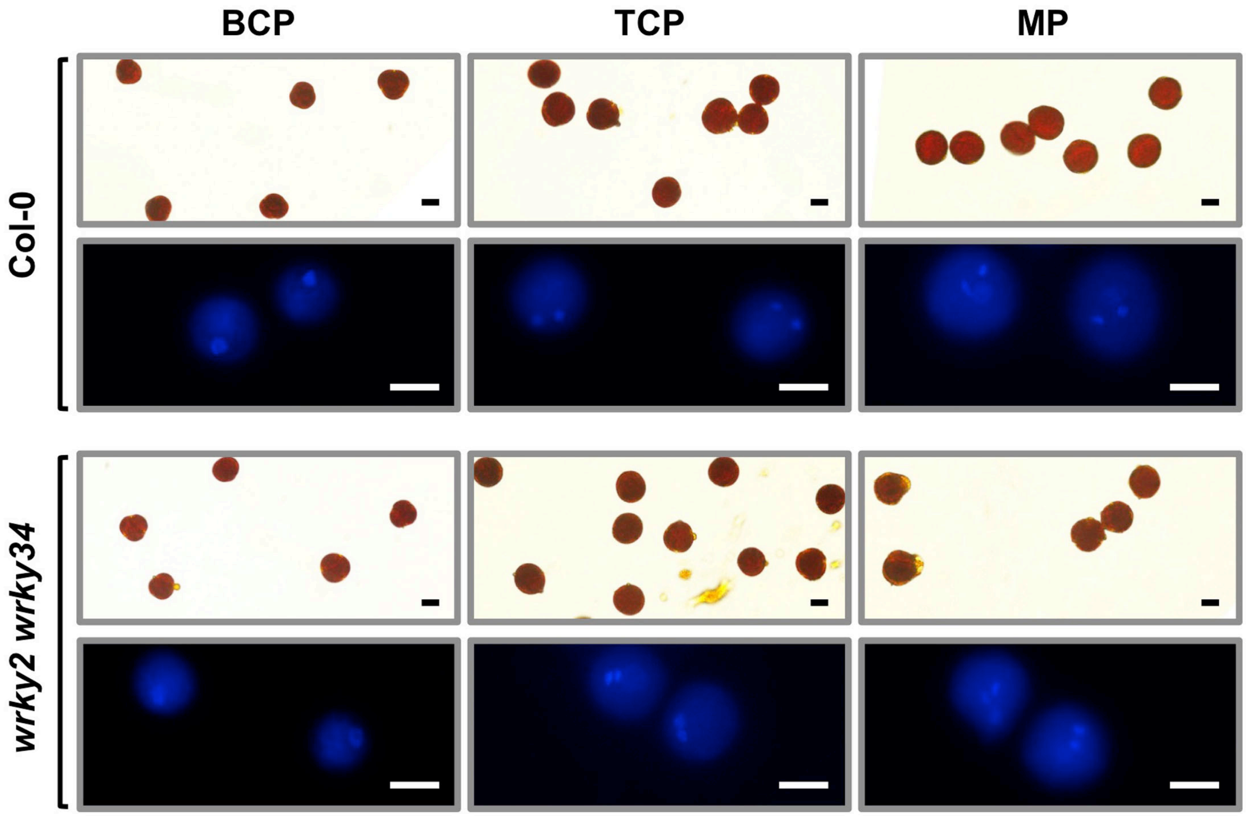

Supplemental Figure S10. Starch accumulation during pollen development in Col-0 and *wrky2 wrky34* double mutant plants.

Pollen grains from Col-0 and *wrky2 wrky34* plants were stained with Lugol's iodine solution and imaged. Top panels: Lugol's iodine staining to show starch accumulation; and bottom panels: DAPI staining of pollen grains from the same anthers to determine pollen nuclear stage. BCP, bicellular pollen; TCP, tricellular pollen; and MP, mature pollen. Bar = 10 µm.
