# Supplementary material for: Regulation of pollen lipid body biogenesis by MAP kinases and downstream WRKY transcription factors in Arabidopsis
Source: PLoS Genet. 2018 Dec 26;14(12):e1007880. doi: 10.1371/journal.pgen.1007880 (PMC6324818; doi:10.1371/journal.pgen.1007880)
Supplement: S11 Fig — (A) BODIPY 505/515 staining of lipid bodies in pollen grains from wrky2 wrky34 and PLAT52:GPT1-eYFP wrky2 wrky34 plants. (B) Quantitation of BODIPY 505/515 fluorescence intensity in pollen grains from wrky2 wrky34 and PLAT52:GPT1-eYFP wrky2 wrky34 plants. Fluorescence intensity was quantified using ImageJ and normalized to that in wrky2 wrky34, which was set as 100%. Three independent PLAT52:GPT1-eYFP transgenic lines in wrky2 wrky34 background were analyzed and all gave similar results. Results from one of the three lines are shown. Error bars indicate SD (n ≥ 35). **P ≤ 0.01. Bar = 10 μm. (PDF) [file pgen.1007880.s013.pdf]

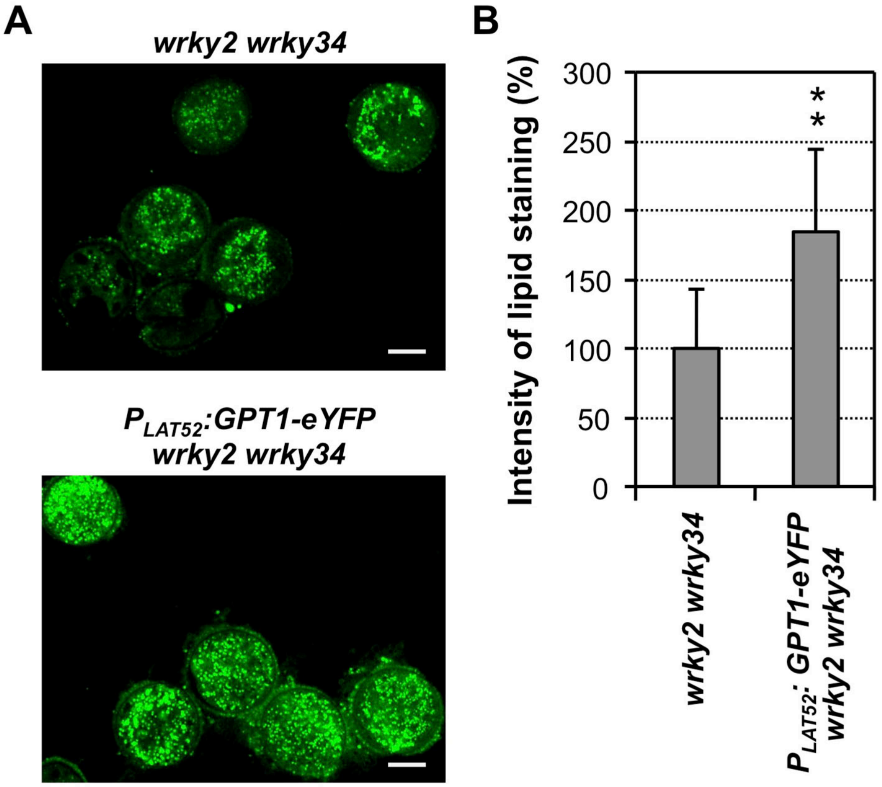

Supplemental Figure S11. Pollen-specific overexpression of *GPT1-eYFP* enhances the accumulation of lipid bodies in *wrky2 wrky34* pollen.
(**A**) BODIPY 505/515 staining of lipid bodies in pollen grains from *wrky2 wrky34* and P_{LAT52}*:GPT1-eYFP wrky2 wrky34* plants. (**B**) Quantitation of BODIPY 505/515 fluorescence intensity in pollen grains from *wrky2 wrky34* and P_{LAT52}*:GPT1-eYFP wrky2 wrky34* plants. Fluorescence intensity was quantified using ImageJ and normalized to that in *wrky2 wrky34,* which was set as 100%. Three independent P_{LAT52}*:GPT1-eYFP* transgenic lines in *wrky2 wrky34* background were analyzed and all gave similar results. Results from one of the three lines are shown. Error bars indicate SD ($n \geq 35$). **$P \leq 0.01$. Bar = 10 μm.
