# Supplementary material for: Regulation of pollen lipid body biogenesis by MAP kinases and downstream WRKY transcription factors in Arabidopsis
Source: PLoS Genet. 2018 Dec 26;14(12):e1007880. doi: 10.1371/journal.pgen.1007880 (PMC6324818; doi:10.1371/journal.pgen.1007880)
Supplement: S12 Fig — (A) Four W-boxes in the GPT1 promoter were combined to a 106-nucleotide-long GPT1 promoter fragment (PGPT1), which was used for DNA-binding assay in yeast. W-boxes are marked in black. Red-colored letters indicate mutated nucleotides in the W boxes of GPT1 promoter fragment (mPGPT1). (B) Yeast was co-transformed with a reporter vector containing the promoter fragment of PGPT1 or mPGPT1 fused to a HIS2 reporter gene, and an effector vector containing WRKY34 fused to a GAL4 activation domain. Transformants were selected on double dropout medium (SD-Leu-Trp) and then plated on triple dropout medium (SD-Leu-Trp-His) to test binding. 3-amino-1, 2, 4-triazole (3-AT, 90 mM) was included to suppress background growth. (PDF) [file pgen.1007880.s014.pdf]

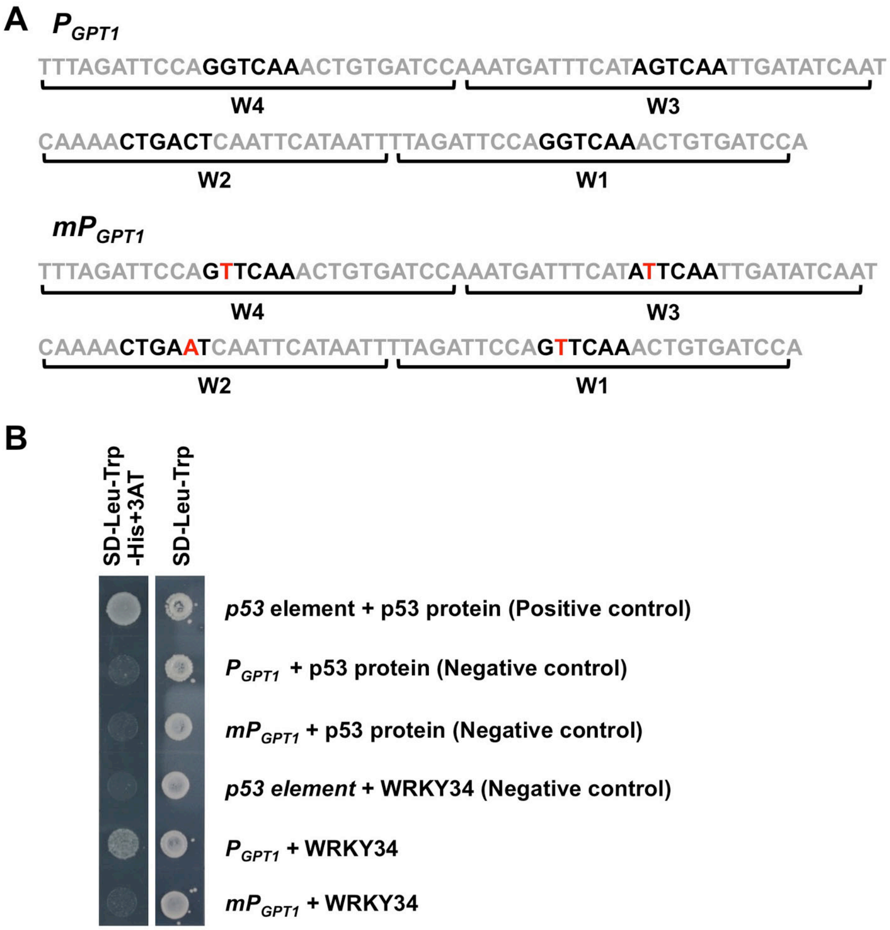

Supplemental Figure S12. WRKY34 binds to *GPT1* promoter in yeast one-hybrid assay. (**A**) Four W-boxes in the *GPT1* promoter were combined to a 106-nucleotide-long *GPT1* promoter fragment (P_{GPT1}), which was used for DNA-binding assay in yeast. W-boxes are marked in black. Red-colored letters indicate mutated nucleotides in the W boxes of *GPT1* promoter fragment (mP_{GPT1}). (**B**) Yeast was co-transformed with a reporter vector containing the promoter fragment of P_{GPT1} or mP_{GPT1} fused to a *HIS2* reporter gene, and an effector vector containing WRKY34 fused to a GAL4 activation domain. Transformants were selected on double dropout medium (SD-Leu-Trp) and then plated on triple dropout medium (SD-Leu-Trp-His) to test binding. 3-amino-1, 2, 4-triazole (3-AT, 90 mM) was included to suppress background growth.
