# Supplementary material for: Regulation of pollen lipid body biogenesis by MAP kinases and downstream WRKY transcription factors in Arabidopsis
Source: PLoS Genet. 2018 Dec 26;14(12):e1007880. doi: 10.1371/journal.pgen.1007880 (PMC6324818; doi:10.1371/journal.pgen.1007880)
Supplement: S13 Fig — (A) BODIPY 505/515 staining of lipid bodies in pollen grains from Col-0, mkk4, mkk5, and mkk4 mkk5 plants. (B) Quantitation of BODIPY 505/515 staining of pollen grains from Col-0, mkk4, mkk5, and mkk4 mkk5 plants. The intensity of fluorescence was quantified using ImageJ, and normalized to that in Col-0 control, which was set as 100%. Error bars indicate SD (n ≥ 20). **P ≤ 0.01. Bar = 10 μm. (PDF) [file pgen.1007880.s015.pdf]

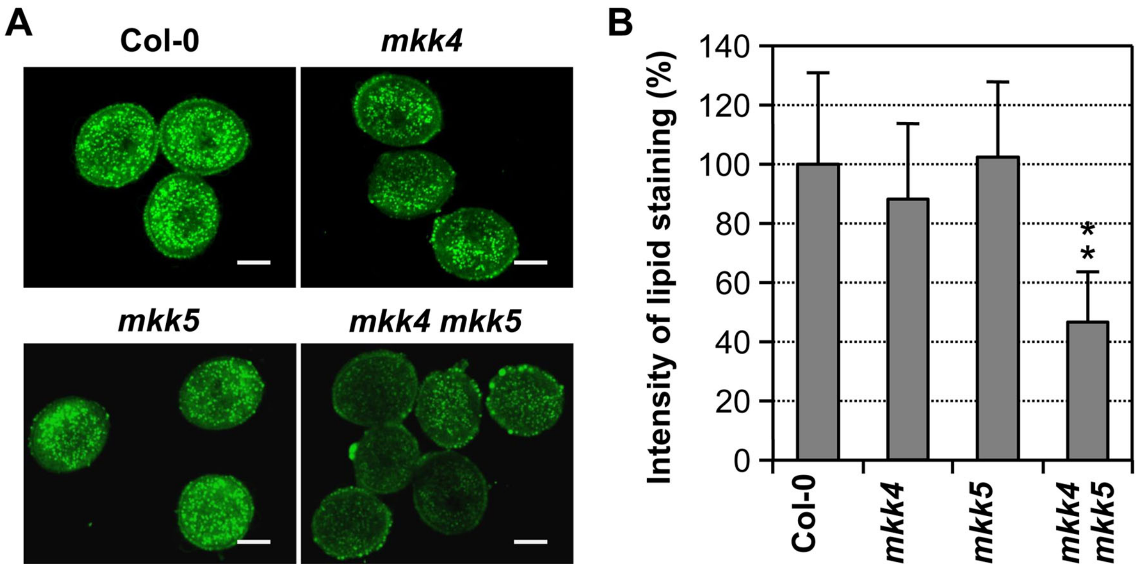

Supplemental Figure S13. Loss of function of both *MKK4* and *MKK5* compromises lipid body accumulation in mature pollen.

(**A**) BODIPY 505/515 staining of lipid bodies in pollen grains from Col-0, *mkk4*, *mkk5*, and *mkk4 mkk5* plants. (**B**) Quantitation of BODIPY 505/515 staining of pollen grains from Col-0, *mkk4*, *mkk5*, and *mkk4 mkk5* plants. The intensity of fluorescence was quantified using ImageJ, and normalized to that in Col-0 control, which was set as 100%. Error bars indicate SD ($n \geq 20$). $^{**}P \leq 0.01$. Bar = 10 μm.
