# Supplementary material for: Regulation of pollen lipid body biogenesis by MAP kinases and downstream WRKY transcription factors in Arabidopsis
Source: PLoS Genet. 2018 Dec 26;14(12):e1007880. doi: 10.1371/journal.pgen.1007880 (PMC6324818; doi:10.1371/journal.pgen.1007880)
Supplement: S14 Fig — (A) BODIPY 505/515 staining of lipid bodies in pollen grains from chemical genetically rescued MPK6SR plants treated with either DMSO solvent or NA-PP1. (B) Quantitation of BODIPY 505/515 fluorescence intensity in pollen grains from DMSO- or NA-PP1-treated MPK6SR plants. The intensity of BODIPY fluorescence was quantified using ImageJ and normalized to that in the DMSO-treated control, which was set as 100%. Error bars indicate SD (n ≥ 25). **P ≤ 0.01. Bar = 10 μm. (PDF) [file pgen.1007880.s016.pdf]

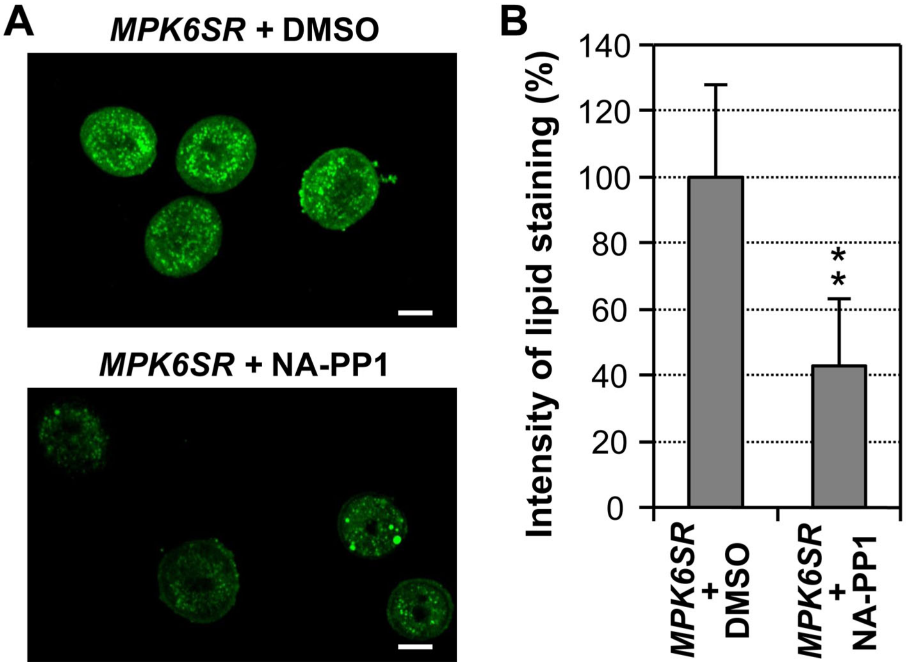

Supplemental Figure S14. Loss of function of *MPK3* and *MPK6* compromises lipid body accumulation in mature pollen.
(**A**) BODIPY 505/515 staining of lipid bodies in pollen grains from chemical genetically rescued *MPK6SR* plants treated with either DMSO solvent or NA-PP1. (**B**) Quantitation of BODIPY 505/515 fluorescence intensity in pollen grains from DMSO- or NA-PP1-treated *MPK6SR* plants. The intensity of BODIPY fluorescence was quantified using ImageJ and normalized to that in the DMSO-treated control, which was set as 100%. Error bars indicate SD ($n \geq 25$). $^{**}P \leq 0.01$. Bar = 10 μm.
